# Simple MD-based model for oxidative folding of peptides and proteins

**DOI:** 10.1038/s41598-017-09229-7

**Published:** 2017-08-24

**Authors:** Sergei A. Izmailov, Ivan S. Podkorytov, Nikolai R. Skrynnikov

**Affiliations:** 1 Laboratory of Biomolecular NMR, St. Petersburg State University, St. Petersburg, 199034 Russia; 2 Department of Chemistry, Purdue University, West Lafayette, IN 47907 USA

## Abstract

Significant strides have been recently made to fold peptides and small proteins *in silico* using MD simulations. However, facilities are currently lacking to include disulfide bonding in the MD models of protein folding. To address this problem, we have developed a simple empirical protocol to model formation of disulfides, which is perturbation-free, retains the same speed as conventional MD simulations and allows one to control the reaction rate. The new protocol has been tested on 15-aminoacid peptide guanylin containing four cysteine residues; the net simulation time using Amber ff14SB force field was 61 μs. The resulting isomer distribution is in qualitative agreement with experiment, suggesting that oxidative folding of guanylin *in vitro* occurs under kinetic control. The highly stable conformation of the so-called isomer 2(B) has been obtained for full-length guanylin, which is significantly different from the poorly ordered structure of the truncated peptide PDB ID 1GNB. In addition, we have simulated oxidative folding of guanylin within the 94-aminoacid prohormone proguanylin. The obtained structure is in good agreement with the NMR coordinates 1O8R. The proposed modeling strategy can help to explore certain fundamental aspects of protein folding and is potentially relevant for manufacturing of synthetic peptides and recombinant proteins.

## Introduction

*In silico* folding of protein structures is a Holy Grail of computational modeling. Early efforts were focused on helical and β-hairpin peptides, as well as mini-proteins^1–3^, and often relied on enhanced sampling schemes^4, 5^. More recently, technological advances at D. E. Shaw Research made it possible to reproduce the folding of several small proteins in conventional (unbiased) MD simulations^6–8^. New enhanced sampling schemes have also evolved to fold small proteins^9, 10^.

The existing MD models of protein folding, however, do not have any facilities to include the effect of oxidative folding, i.e. primarily formation of disulfide bonds. Initially, protein dynamics leading to disulfide formation has been modeled using empirical lattice models of different flavor^11–13^. Subsequently, Martí-Renom and Karplus proposed the constrained MD algorithm where two thiols were adiabatically steered toward each other during the course of the short trajectory^14^. The method is elegant and simple to implement; it has been successfully used by the developers to recover small target structures. However, the closure of the disulfide bond in this algorithm is essentially a forced process (inevitable compromise given the limited computational resources available at that time), which is inconsistent with the natural course of polypeptide folding. Specialized coarse-grained force fields have also been developed by Scheraga and others to address the problem of oxidative protein folding. Initially these force fields were used in conjunction with simulated annealing algorithms^15, 16^ and later employed in the context of *bona fide* MD simulations^17, 18^. While useful for large proteins and protein assemblies with a complex architecture, this method does not lead to atomic-resolution models.

Thus, the current situation is such that unbiased MD simulations can be used to successfully fold a (cysteine-free) 76-residue protein ubiquitin^6^, but lack the facilities to attempt folding of another popular model protein, 58-residue BPTI, which contains three disulfide bridges^19^. Our paper describes a simple empirical approach to address this deficiency.

## Results and Discussion

### General aspects of disulfide formation

It has been fully appreciated that disulfide bond formation, as normally occurs in the oxidative environment of the endoplasmic reticulum (ER), is one of the major determinants of protein folding^20–23^. In many situations disulfide bonding can be viewed as a driver of protein folding^24, 25^. Furthermore, it often holds key to protein misfolding and aggregation^26, 27^. It is also clear that oxidative folding in the ER is a highly complicated process, which involves an assortment of thiol-disulfide oxidoreductases with oxidizing or reducing redox potentials (including disulfide isomerases), chaperones, redox-active small molecules, metal ions, etc. operating in the dense lumen environment.

Computational modeling of the ER machinery is well beyond the limits of conventional MD methodology. However, it is feasible to model the folding of peptides and smaller proteins *in vitro*. For instance, one can hope to produce meaningful models for oxidative folding of peptides under exposure to oxygen of air or hydrogen peroxide (i.e. small polar molecules with relatively free access to thiol sites). Such models should offer additional options in our quest for understanding the fundamental biophysics of protein folding. In addition, they are also relevant for recombinant proteins and synthetic peptides^28–30^.

A good example is provided by insulin. Modern synthetic protocols to manufacture insulin rely on orthogonal protective groups to mask thiol sites and thus achieve the site-directed disulfide bonding^31^. Nonetheless, the key step in this process remains the oxidation and bonding of free thiols. Of note, a number of mutations have been identified in proinsulin that interfere with cysteine alignment; these mutations are clinically relevant in the context of neonatal-onset diabetes mellitus^32^. Oftentimes, the effect of these mutations cannot be easily rationalized on the basis of the native structure – instead, it appears that these mutations affect folding intermediates on the path toward the mature insulin. A problem like this would be well suited for MD studies using today’s powerful computers.

One-electron oxidants, such as ${{\rm{O}}}_{2}^{\bullet -}$, can convert cysteine thiolates −S^−^ into thiyl radicals −S^•^. Under aerobic conditions, these radicals can react with other thiolates to form disulfide bridges:^33, 34^

1 $${{\rm{R}}}_{1}\mbox{--}{{\rm{S}}}^{\bullet }+{{\rm{R}}}_{2}\mbox{--}{{\rm{S}}}^{-}+{{\rm{O}}}_{2}\to {{\rm{R}}}_{1}\mbox{--}{\rm{S}}\mbox{--}{\rm{S}}\mbox{--}{{\rm{R}}}_{2}+{{\rm{O}}}_{2}^{\bullet -}$$

While this mechanism does not appear to be biologically relevant, it has been established *in vitro* ^35^.

The mechanism that appears to be highly relevant *in vivo* involves two-electron oxidants, such as H_2_O_2_. This route involves first the formation of sulfenic acid intermediate, which subsequently reacts with thiolate to form a disulfide bond:^34, 36^

21 $${{\rm{R}}}_{1}\mbox{--}{{\rm{S}}}^{-}+{{\rm{H}}}_{2}{{\rm{O}}}_{2}\to {{\rm{R}}}_{1}\mbox{--}{\rm{SOH}}+{{\rm{OH}}}^{-}$$

22 $${{\rm{R}}}_{1}\mbox{--}{\rm{SOH}}+{{\rm{R}}}_{2}\mbox{--}{{\rm{S}}}^{-}\to {{\rm{R}}}_{1}\mbox{--}{\rm{S}}\mbox{--}{\rm{S}}\mbox{--}{{\rm{R}}}_{2}+{{\rm{OH}}}^{-}$$

**Figure 1:**
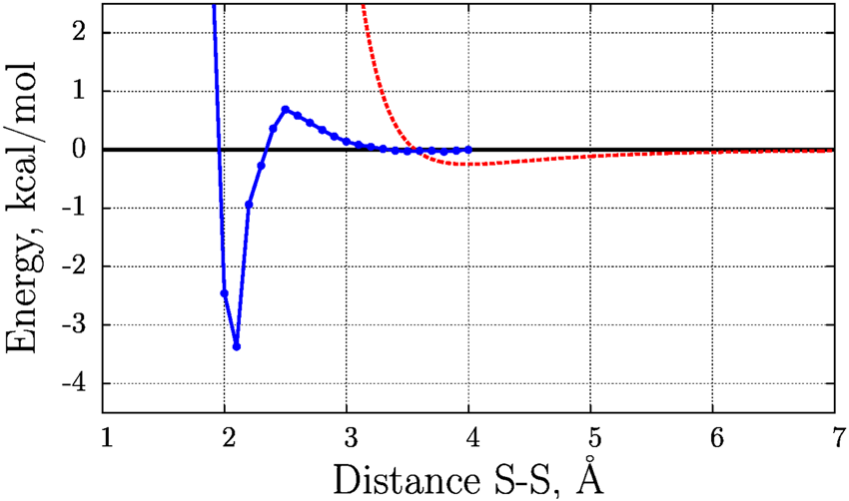
*Blue curve*: potential energy scan with respect to the sulfur-sulfur distance for the reaction between CH_3_−SOH and CH_3_−S^−^. Reproduces the original result by Kassim *et al*.^38^ calculated at the mPW1PW91/6-311 + G(d,p) level of theory with the polarizable continuum model. *Red dashed curve*: Lennard-Jones potential $\varepsilon ({({r}_{0}/r)}^{12}-2{({r}_{0}/r)}^{6})$ for a pair of cysteine sulfur atoms as parameterized in the Amber 14SB force field, *r*_0_ = 4 Å and *ε* = 0.25 kcal/mol. This pairwise sulfur-sulfur potential is disabled in the described model of oxidative folding.

Similar mechanism, involving sulfenic acid intermediate, has been demonstrated for disulfide bonding of cysteins exposed to O_2_ of air in the presence of trace metals^37^. The energy barrier of reaction (2.2) has been evaluated by Kassim *et al*. for methanesulfenic acid and methanethiolate (blue profile in Fig. 1)^38^. At 0.7 kcal/mol, this barrier is low. Given the limited accuracy of the DFT method employed in these computations, one may view this reaction as essentially barrierless.

Another pathway that is worth mentioning in this context is thiolate-disulfide exchange reaction:^39^

3 $${{\rm{R}}}_{1}\mbox{--}{\rm{S}}\mbox{--}{\rm{S}}\mbox{--}{{\rm{R}}}_{2}+{{\rm{R}}}_{3}\mbox{--}{{\rm{S}}}^{-}\to {{\rm{R}}}_{1}\mbox{--}{\rm{S}}\mbox{--}{\rm{S}}\mbox{--}{{\rm{R}}}_{3}+{{\rm{R}}}_{2}\mbox{--}{{\rm{S}}}^{-}$$

Note also that disulfide bond, as well as sulfenic acid, can be reduced to the thiol form by application of dithiothreitol^40^.

All of the above mechanisms favor thiolate form over thiol; therefore, their efficiency is highly sensitive to cysteine pKa (the more nuanced discussion of the pKa dependence can be found elsewhere^41, 42^). More generally, in the context of structured proteins the efficiency of these reactions can be greatly influenced by electrostatic and steric factors and vary by many orders of magnitude^43^.

### MD model of disulfide formation

In our MD model, we introduce cysteine residues that contain deprotonated thiols, but remain overall neutral. The residue type defined in this manner is termed CYR. One can think of CYR side chain as thiyl radical $-{{\rm{S}}}^{\bullet }$. A pair of such radicals can react in a barrierless fashion to produce a disulfide bond. It should be mentioned, however, that this mechanism has little direct relevance since thiyl radicals are extremely short-lived and therefore a close encounter between the two radicals is unlikely^44, 45^. Alternatively, one can view free, deprotonated, neutral cysteines (−S) as mimetics of cysteine sulfenate (−SOH) and cysteine thiolate anion (−S^−^), which are capable of disulfide bonding according to Eq. (2.2). This latter interpretation can raise questions and therefore needs to be addressed in more detail.

First, we notice that the substitution of −S for −SOH or −S^−^ is akin to a point mutation. While some point mutations can be highly perturbing, in most cases they leave protein structure intact and folding characteristics largely unchanged. Below we demonstrate that a peptide carrying −SH thiols behaves similar to the −S variant. Likewise, the appearance of −SOH or −S^−^ in the sequence is not expected to strongly alter the dynamics of an unfolded peptide chain or the pattern of cysteine-cysteine contacts compared to −SH or −S variants.

Second, it should be pointed out that such issues are, in fact, frequently encountered (and routinely ignored) in protein MD simulations. The difference between −SH and −S^−^ is the same as the difference between neutral and charged form of other titratable amino acids, e.g. histidine, glutamic acid, or aspartic acid. If pK_a_ of a certain residue happens to be close to the nominal pH of the MD simulation, then modeling of such site inevitably becomes problematic (unless one resorts to specialized techniques such as constant-pH Molecular Dynamics^46–48^). The problem is especially significant for folding simulations where pK_a_ could, in principle, vary along the folding pathway. However, such potential complications have not prevented successful recent efforts in the area of protein folding.

Third, we believe that there is no practical alternative to the simple model such as employed here. If one were to pursue a more rigorous approach, one would need to model not one, but two chemical reactions, (2.1) and (2.2), where the first step involves a small oxidant molecule. The orientational dependence of both reactions is unknown and, in any event, it would not be a trivial matter to incorporate such orientational dependence into an MD protocol. For those peptides and proteins that involve more than two cysteines (which are the most interesting targets for studying oxidative folding) such simulations can become exceedingly cumbersome. Indeed, multiple series of simulations would be needed to model all possible combinations of cysteine species (with one cysteine in −SOH form, another one in −S^−^ form, and all others in the −SH state). Because it is currently impossible to accurately and efficiently incorporate chemical reactions into folding simulations, any benefits from explicit −SOH and −S^−^ modeling would be forfeited.

Finally, the ultimate criterion is the ability of the simplified model to obtain correct peptide or protein folds. As shown below, this can be successfully accomplished using the proposed MD protocol.

In what follows we outline the procedure used toward this goal. For simplicity, let us assume that MD simulation begins with a random-conformation peptide that contains reactive CYR cysteines. The trajectory is recorded in a standard fashion, with one single exception. Specifically, we switch off the Lennard-Jones (LJ) potential between pairs of sulfurs belonging to CYR residues. This allows the two sulfurs to move within capture radius of each other and form a bond. Our definition of the capture radius is informed by the data in Fig. 1 (blue profile), *r*_*c*_ = 2.5 Å. Once the two sulfur atoms diffuse within 2.5 Å of each other during the course of MD simulation, the procedure is initiated to form a disulfide bond.

A special effort has been made to ensure that “bond formation” does not perturb the simulation. For this purpose we first introduce artificial restraints that emulate the disulfide bond with reduced strength. Specifically, these are harmonic restraints involving the S-S distance, C-S-S and S-S-C bond angles, and the set of dihedral angles associated with disulfide bridge (see Table S1 for complete list). Their initial strength is set to 1/200 of the respective force field parameters. The restraints are then progressively ramped up until they reach the target values. This is done in a step-wise manner over the time interval of 2 ns. As a result the sulfur atoms are gently pulled to the final distance 2.038 Å (which is the equilibrium length of the disulfide bond). Similarly, the bond angles are softly steered toward their target values 103.7° and the dihedral angles are gradually restrained as appropriate. During this process we have not observed any interference from outside atoms, i.e. no intervening water molecules, etc.

Once the disulfide bond is effectively established in this manner, the user-defined restraints are removed and replaced with the (equivalent) generic restraints, which represent the disulfide bond in the chosen force field. Specifically, the topology file is regenerated to reflect the newly formed disulfide bridge; in doing so the two bonded CYR residues are converted to standard disulfide-linked CYX type. The simulation is then continued until all disulfide bridges are formed (or, if desired, beyond this point). Of note, the algorithm has been implemented essentially without any loss of speed compared to conventional MD run. Monitoring sulfur-sulfur distances causes only 0.6% loss in the speed of computations; additional expense to make disulfide bonds do not exceed several nanoseconds of the simulation time.

It should be emphasized that essentially at all time the simulations are conducted under the control of the original, unaltered force field. The exceptions are brief periods of time when CYR sulfur atoms approach each other to within less than 3.5 Å. In this situation, there is a distinction – in our MD model, there is no LJ repulsion between the two sulfur atoms (see Fig. 1) and, therefore, they can further approach each other. If the distance 2.5 Å is ever reached, then the procedure to form a disulfide bridge is launched; otherwise the two cysteines drift apart and the simulation continues in a conventional manner.

In general terms, the process of oxidative folding is governed by two factors: (*i*) conformational dynamics of polypeptide chain and (*ii*) the efficiency of chemical reaction leading to disulfide bonding. The fundamental hypothesis in our model is that this dependence is *factorizable* – i.e. we can reasonably well reproduce the dependence on conformational dynamics, while making certain simplistic assumptions about chemistry.

For instance, in the context of a disordered peptide chain, it can be assumed that disulfide bonding occurs in the environment that can be described as “generic”, i.e. subject to dynamic averaging, featureless, and generally neutral toward the progress of the reaction. (This is in contrast to redox enzymes, where the active-site environment is tailored to facilitate the progress of the reaction.) In the case of disordered proteins or peptides, we can assume that the rate of disulfide formation is constant – e.g. each close encounter between the cysteine thiol groups leads to a formation of S-S bond, irrespective of the identity of the cysteines involved or the instantaneous configuration of the surrounding side chains. In this manner the disulfide bonding chemistry is essentially “factored out” in our simplified treatment.

This approach also allows one to accomplish an oxidative folding of a peptide (or a small protein) on a time scale of an MD simulation. That is many orders of magnitude faster than normally observed *in vitro*, where the experiments are typically conducted with modest oxidant concentrations. If necessary, it is possible to adjust the rate of disulfide bonding in our MD model so as to balance it against other drivers of folding, such as hydrophobic interactions. For example, it can be requested that only a certain fraction of close encounters between the cysteine thiol groups is productive and results in a formation of a disulfide bond. In what follows we demonstrate such amended algorithm.

### Guanylin simulation

**Figure 2:**
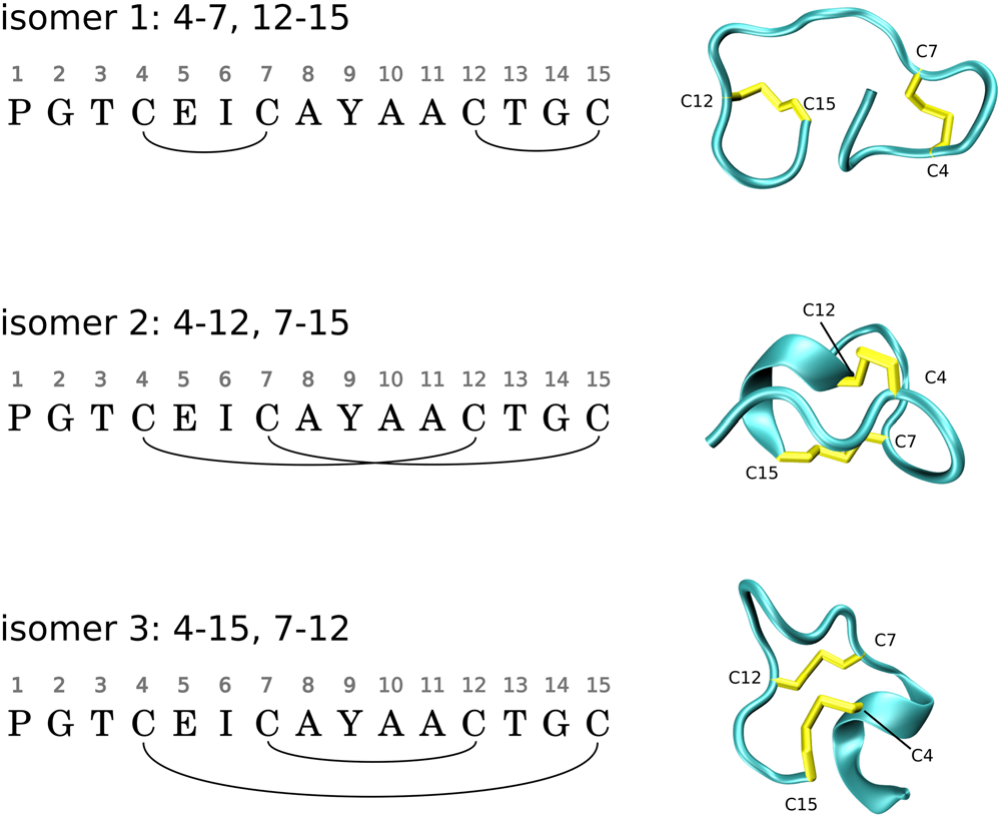
Three isomers of guanylin, including the biologically active form, isomer 2. The MD frames are shown for illustration purpose.

Guanylin regulates the electrolyte balance in the intestine via interaction with guanylate cyclase C receptor^49^. A mature guanylin is a peptide consisting of 15 amino acids, including four cysteine residues. Different pairwise disulfide patterns correspond to three isomers of guanylin. All these isomers have been obtained and characterized *in vitro*, although only one of them is physiologically relevant (see Fig. 2)^50^. Of interest, guanylin controls proliferation of epithelial cells and possesses tumor-suppressor properties^51, 52^. Certain bacterial enterotoxins bear close resemblance to guanylin and thus interact with guanylate cyclase C receptor; this is the main cause of dehydration effects associated with diarrhea^53, 54^.

To simulate oxidative folding of guanylin, we begin by generating 100,000 random guanylin conformations. For this purpose we have used the software by Sosnick *et al*.^55^ (unfolded.uchicago.edu) augmented with the program Scwrl4^56^. To evaluate the energy distribution of the resulting ensemble, we have used the sander program from AmberTools with Amber ff14SB force field and implicit solvent (option igb = 8)^57, 58^. To a good approximation, the Gaussian distribution in energy has been obtained, as expected for the valid conformational ensemble (see Fig. S1A)^59, 60^.

Each of the 100,000 random conformations of guanylin has been solvated by building a truncated octahedron box with the 5 Å spacing between the peptide and the boundary. The resulting distribution of box sizes is illustrated in Fig. S1B. The *maximum* box size was found to be 56 Å (the diameter of the inscribed sphere). This very large box size, accommodating the most extended guanylin conformations, was chosen for all subsequent simulations so as to eliminate any potential bias due to influence of the periodic boundary conditions on peptide folding.

**Figure 3:**
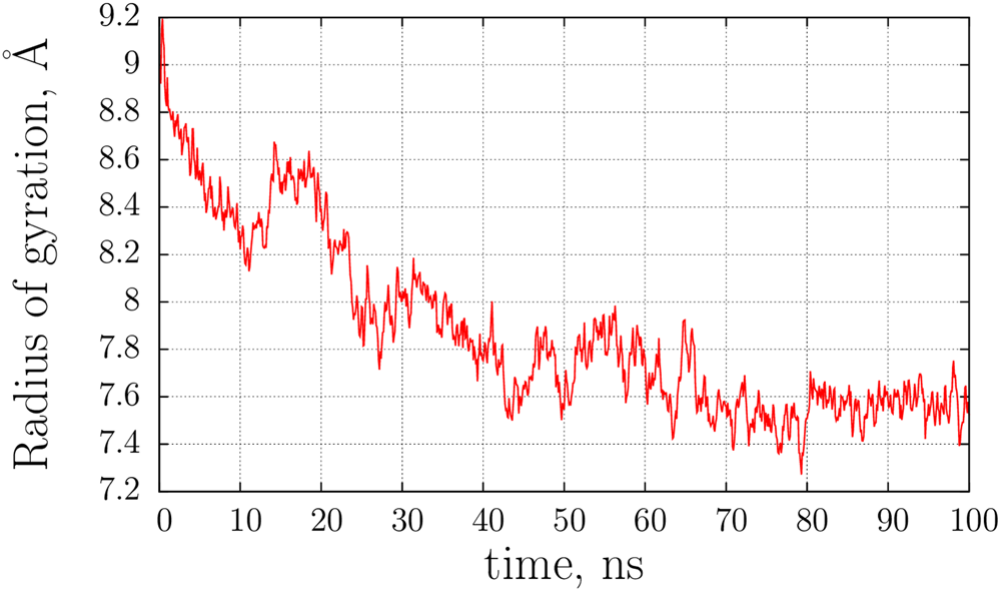
Radius of gyration of disordered (reduced) guanylin during the first 100 ns of MD simulation (averaged over 50 trajectories).

As a next step, 50 guanylin conformations were chosen randomly from the initial pool of structures and then solvated with TIP3P water using the fixed cell size 56 Å, thus producing the systems with ca. 11,600 atoms. Each system was then evolved for 100 ns in Amber 14 (ff14SB). At this stage, all cysteine residues have been assigned to the type CYR; otherwise this is a conventional simulation with the standard set of force field parameters and no special facilities to form disulfide bonds. During this time period the initial ensemble, comprised of the randomly built conformers, evolves into the structurally equilibrated state. The convergence can be conveniently monitored through the time dependence of the radius of gyration. As shown in Fig. 3, the average *R*_*g*_ value, calculated across the set of 50 trajectories, is initially 9 Å. Over the course of the simulation it gradually decreases until reaching a plateau at ca. 7.6 Å.

The decline in average *R*_*g*_ occurs over the time interval of several tens of nanoseconds. As it appears, we observe here a “prefolding” process, where a number of conformational rearrangements take place, leading the system toward the favorable conformational basin. It is worth mentioning that partial compaction of the peptide may be dependent on the details of the force field and/or the water model^61, 62^. Of note, the use of CYR residues during this stage did not influence the outcome of the simulations. To confirm this, we have additionally recorded fifty 100-ns control trajectories of guanylin containing standard CYS residues. These control simulations produced a conformational ensemble with the same average radius of gyration and the same distribution of sulfur-to-sulfur distances, see Fig. S2.

**Figure 4:**
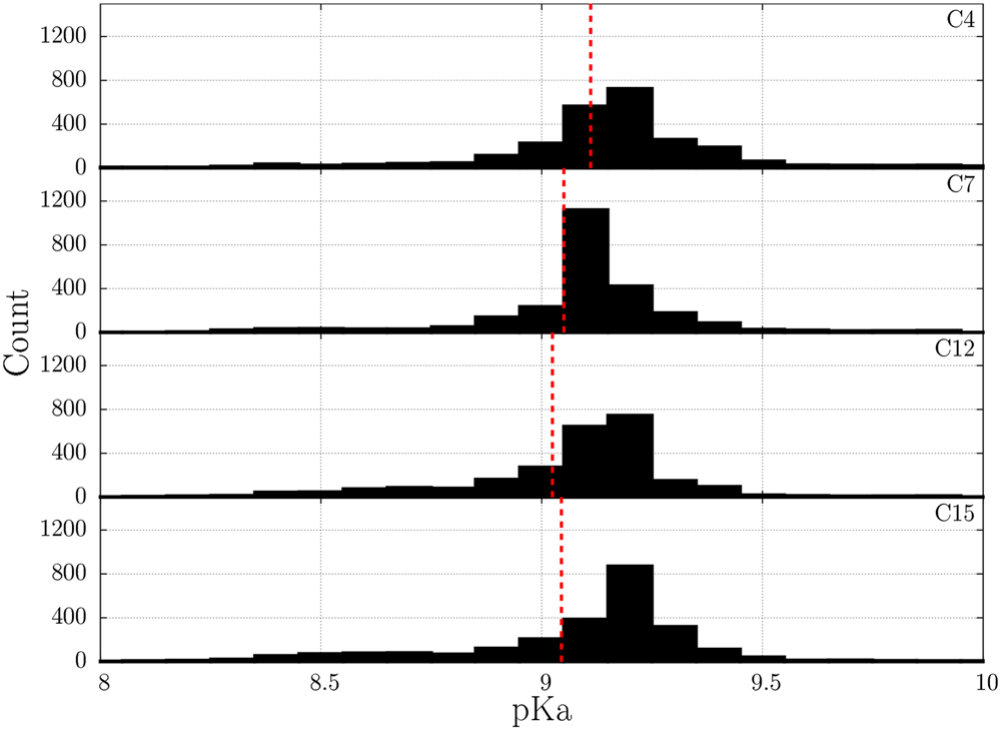
Distribution of the cysteine thiol pKa values in MD simulation of guanylin as calculated with the program PROPKA (50 trajectories, 50 frames per trajectory). The mean pKa values are indicated with vertical red lines. As an aside, mean pKa values serve only the purpose of comparison. Based on the data at hand, it is not possible to determine an effective pKa which would be representative of the average concentration of the reactive −S^−^ species. In fact, such effective pKa for a peptide rapidly interconverting between different conformations should depend on the cysteine protonation/deprotonation rates.

The converged portion of the simulation, from 50 to 100 ns, was used to calculate the cysteine thiol pKa values. Toward this goal, we have applied the program PROPKA 3.1^63^ to a series of snapshots extracted from the 50 guanylin trajectories. The results are presented in Fig. 4. For all four cysteine residues the data are in line with expectations (generally, thiol pKa of the fully solvated cysteine side chain is ca. 8.8;^64^ for free glutathione the value is 9.2^65^). All cysteines in guanylin display very similar mean pKa values, with pairwise deviations less than 0.1 units (between C12 and C4).

This is a significant observation in the light of the above discussion on the mechanisms of the disulfide bond formations. The thiol pKa is a major determinant of all disulfide-forming reactions Eqs (1–3). The fact that the calculated pKa values in all cysteine residues are similar implies that the reaction rates for all pairs of cysteines are similar. This paves the way to the simple model of oxidative folding as described above.

Fundamentally, the observed similarity of cysteine pKa values supports our conjecture that the problem is factorizable, i.e. that modeling of the peptide dynamics can be separated from the treatment of the chemical reaction. Furthermore, the rate of the chemical reaction can be used effectively as a scaling parameter. Under mildly oxidizing conditions, as typically employed in experimental peptide studies^66^, it can be crudely estimated that 1 in ca. 10^10^ close encounters between cysteine side chains is productive (i.e. leads to disulfide bond formation). On the other hand, in our MD model we postulate that every such encounter is productive (alternatively, we assume that only a fraction of all encounters is productive, see below). In this manner we reproduce the native-like oxidative folding of the peptide in a fraction of the time that this process requires *in vitro*.

### Oxidative folding of guanylin

**Figure 5:**
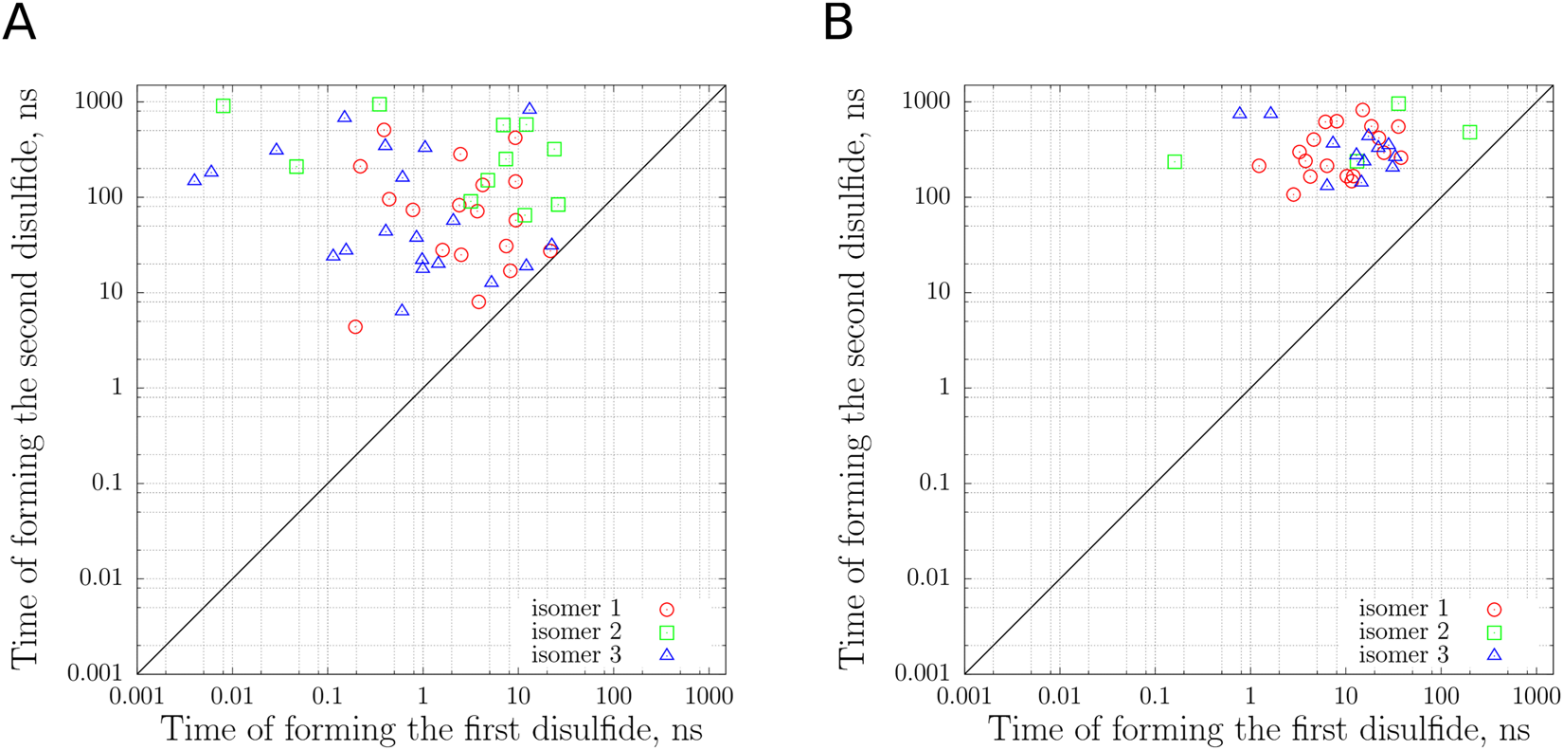
Time points when disulfide bridges were formed in guanylin simulations (first disulfide −*x*-axis, second disulfide – *y*-axis) in (**A**) 49 trajectories where each thiol encounter was treated as productive and (**B**) 34 trajectories with the reduced reaction rate (see text). Different isomers of guanylin (see Fig. 2) are indicated by different symbols as listed in the legend.

Following the 100 ns equilibration period, we have started the MD procedure to model oxidative folding in guanylin. At this stage we switch off pairwise LJ interactions between SG atoms from CYR residues, thus facilitating close encounters between those atoms. Once two sulfur atoms approach each other to within 2.5 Å, the procedure is launched to form a disulfide bridge. Shown in Fig. 5A is the kinetic footprint of disulfide bonding. As can be seen from the plot, the first disulfide bond usually forms relatively quickly – at most, it takes 26 ns. In several cases the first disulfide is formed in less than 10 ps. The second disulfide bond typically takes much longer to form than the first one, on average 198 ns vs. 5 ns. Clearly, the presence of the first bridge sharply reduces the conformational freedom of the peptide – the conformations consistent with both disulfides are rare compared to those that are compatible with a single disulfide. We have also observed the scenario where N-terminal segment of guanylin adopted a β-bridge conformation, which prevented C4 and C7 from forming a disulfide bond for an extended period of time. In fact, one trajectory has not formed the second disulfide bridge at all during the 1 μs oxidative folding simulation. This trajectory has been discontinued and excluded from further analyses.

Each of the forty nine successful simulations was continued beyond the point where the second disulfide was formed. At that stage all four cysteine residues are converted to the standard disulfide-bonded type CYX, so that the following simulation was strictly a conventional MD simulation. The purpose of this additional simulation period with the duration of 450 ns was to allow the structures to relax and then sample their equilibrium dynamics. With this extension our longest trajectory had a net length of 1.50 μs (comprised of 0.10 μs structural equilibration period, 0.95 μs oxidative folding, and 0.45 μs dynamics in the oxidized state). The net length of all forty nine trajectories generated in this manner was 36.7 μs, of which 9.8 μs represent the active phase (oxidative folding).

As already pointed out, during the oxidative folding the force field sensed by the system is mostly indistinguishable from the original force field. The appreciable deviations occur only when two cysteine sulfur atoms approach each other to within 3.5 Å (recall that in our model there is no LJ repulsion between these two atoms). Considering the net duration of the oxidative folding simulation, 9.8 μs, we have determined that 96% of that time the system effectively evolves under the control of the standard, unaltered force field. Only 4% of the time the force field can be described as non-standard – either two sulfur atoms approach each other too closely or artificial restraints are in effect to emulate disulfide bonding.

Furthermore, some of the perturbations caused by close encounters between cysteine thiols are insignificant. For example, consider the situation where two thiols brush past each other, passing at a distance of ca. 3 Å. Clearly, this brief event has little influence on peptide dynamics as a whole. On the other hand, if two sulfurs approach within 2.5 Å, which triggers the process of disulfide formation, the consequences for peptide dynamics are profound. However, the same can be said of disulfide bonding in nature – it leads to big changes in peptide dynamics.

Of note, the formation of disulfide bonds does not perturb the temperature, volume, or pressure of the MD simulations. This is illustrated in Fig. S3 for the trajectory that leads to formation of the low-energy isomer 2 of guanylin (see below for details). We have also investigated the time dependence of several parameters which are indicative of peptide folding: number of intramolecular hydrogen bonds, radius of gyration, and MM-GBSA energy (illustrated in Fig. S4). As one may expect, formation of disulfide bridges is not directly synchronized with changes in hydrogen bonding pattern. On the other hand, disulfide bonding is sometimes accompanied by a distinct drop or increase in *R*_*g*_. Of note, emergence of disulfides does not cause any discernible changes in MM-GBSA energy beyond the usual random fluctuations. This is a satisfying result, suggesting that disulfides do not introduce any additional strain into the simulated system.

Oxidative folding of guanylin, as modeled in our MD simulations, results in different pairing of cysteines. The three obtained isomers are illustrated in Fig. 2. The production of the isomers across the set of 49 trajectories is summarized in Table 1 (third column). The simulations suggest that the product mixture is dominated by isomers 1 and 3, with isomer 2 being least populated. This result is in qualitative agreement with the experimental findings by Schulz and co-workers (second column in Table 1)^67^.

**Table 1:** Distribution of guanylin isomers.

	Experiment^a^	MD^b^
Isomer 1	45%	37%
Isomer 2	5%	22%
Isomer 3	50%	41%

^a^Data from Schulz *et al.* ^67^ for air oxidation of guanylin at 20 °C. The experiment relies on a small oxidant molecule (O_2_) with good access to all peptide sites. This is consistent with the basic premises of the proposed MD model of oxidative folding (see above).

^b^The set of 49 MD simulations resulting in fully oxidized guanylin. We have also recorded an additional series of trajectories which produced virtually identical isomer distribution (simulations with adjusted thiol reactivity, as discussed below).

The interpretation of the MD data is fairly straightforward. The type of the isomer in our model is determined by the identity of the first formed disulfide bridge. Among the 49 trajectories, the most frequently occurring first disulfides are C12-C15 and C12-C7 (14 and 12 occurrences, respectively). These two bridges correspond to isomers 1 and 3. Clearly, the conformational ensemble of guanylin, as modeled by our MD simulations, favors those cysteine contacts. On the other hand, the contacts C12-C4 and C15-C7, corresponding to isomer 2, are among the least favored (7 and 4 occurrences, respectively). Thus, Molecular Dynamics broadly reproduces the relative probabilities of cysteine-cysteine encounters among different cysteine pairs. These probabilities are determined by positioning of the cysteines in the peptide chain of guanylin as well as the conformational preferences of guanylin.

### Analysis of guanylin structures

**Figure 6:**
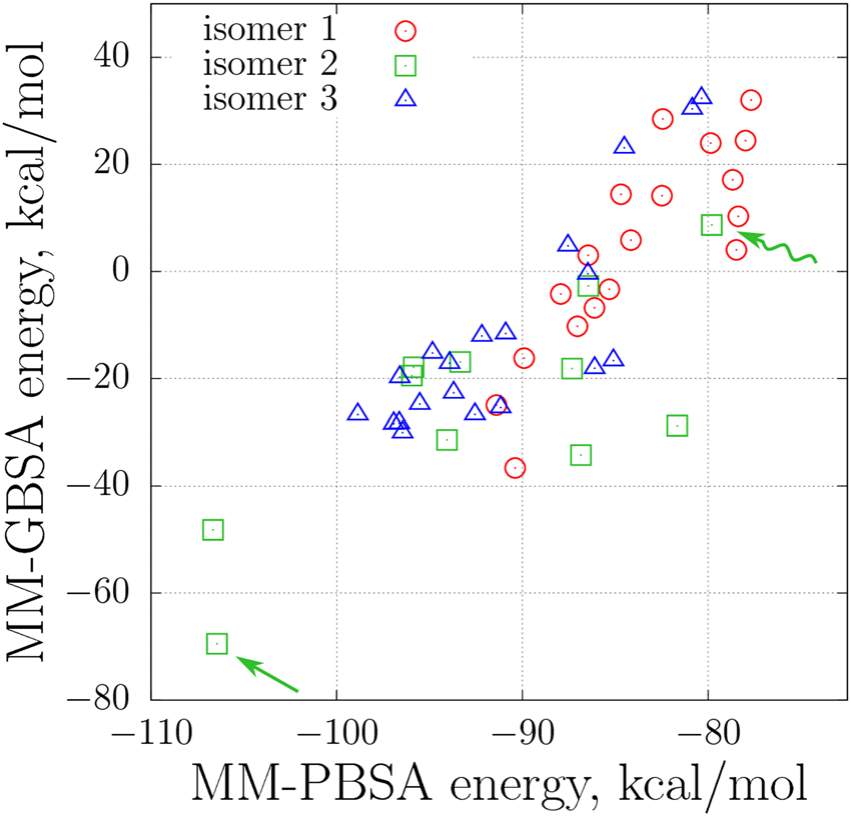
MM-PBSA and MM-GBSA (igb = 8) energies of guanylin as calculated from 49 MD trajectories representing oxidatively folded (two disulfide bonds) guanylin species. The computations were carried out using standard Amber 14 facilities with default set of parameters^58^. Two realizations of isomer 2(B), low-energy model and high-energy 1GNB-like model, are indicated in the plot by the straight and wavy arrows, respectively.

We have used the final 50-ns intervals from all trajectories to assess the quality of the obtained structural models. Toward this goal, MM-PBSA free energies have been calculated for a series of MD frames (sampled with the step 50 ps) and the results were averaged for each individual trajectory. Separately, MM-GBSA energies have been computed in the same manner using igb = 8 option^57^ in Amber 14. The latest GBSA implementations are known to be faster and often more accurate than PBSA, although the results tend to vary from one system to another^68, 69^. In our case we have chosen to use both methods in order to get a better idea of the uncertainty associated with the choice of the algorithm. The results of these calculations are summarized in Fig. 6.

Isomer 1 is least constrained by disulfide bonds (see Fig. 2); as a consequence, it is highly dynamic and tends to be poorly packed. This leads to less favorable free energy values – on average, + 4.2 kcal/mol for isomer 1 vs. –25.3 kcal/mol for isomer 2 and –11.7 kcal/mol for isomer 3 (MM-GBSA data). However, both experimental results and MD simulations indicate that isomer 1 is highly populated. This suggests that isomer 1 can be considered as a kinetically trapped form of guanylin.

Isomer 2 is of special interest in the context of our study. *In vitro* it exists in two different stereoisomeric forms, 2(A) and 2(B), observed to be in 1:1 ratio^70^. Only the former species display biological activity^71^. The two forms interconvert with each other presumably without breaking disulfide bonds, but the exchange is slow, on the time scale of hours^71^. For both forms NMR structures have been solved by Skelton *et al*. (PDB ID 1GNA and 1GNB, respectively)^70^. In our simulations we have obtained 5 realizations of 2(A) and 6 realizations of 2(B), which is consistent with the experimentally observed 1:1 ratio. We have not observed the interconversion between the two forms, which is not surprising given that it occurs on much longer time scale than can be afforded by MD simulations.

In what follows, we discuss the low-energy MD realization of the isomeric form 2(B) (indicated by a straight green arrow in Fig. 6). Focusing on this particular simulation, we observe that toward the end of the trajectory guanylin becomes stabilized in the energetically favorable conformation where it experiences little fluctuations, see Fig. S4. This stable conformation is compact and characterized by a significant number of intramolecular hydrogen bonds, ca. 10. To further evaluate the quality of the MD model, we have selected 20 frames representing the final portion of the trajectory (sampling step 1 ns). The MD ensemble formed in this manner allows for direct comparison with the NMR structure 1GNB, which also consists of 20 conformers. Using the program RAMPAGE^72^, we have found that our MD model contains 84% of residues in the favored regions of the Ramachandran map. This is much better than the statistics for the NMR structure 1GNB, where only 66% of residues belong to the favored regions.

We have also used the well-known validation server WHATIF^73^ to evaluate the packing quality of the two models. The software addresses two questions: (*i*) considering a cluster of residues that are in contact with each other, do they belong together (coarse packing) and (*ii*) assuming that these residues belong together, how well they are packed against each other (fine packing). For the MD ensemble, the two respective scores are −2.2 and −2.1, whereas for the NMR ensemble 1GNB the corresponding scores are −2.7 and −2.8. On WHATIF internal scale, the score of −2.0 corresponds to the high-quality NMR structure, high-quality homology model derived from a good x-ray structure, or low-quality x-ray structure. On the other hand, the score of −3.0 corresponds to a doubtful structure or model.

**Figure 7:**
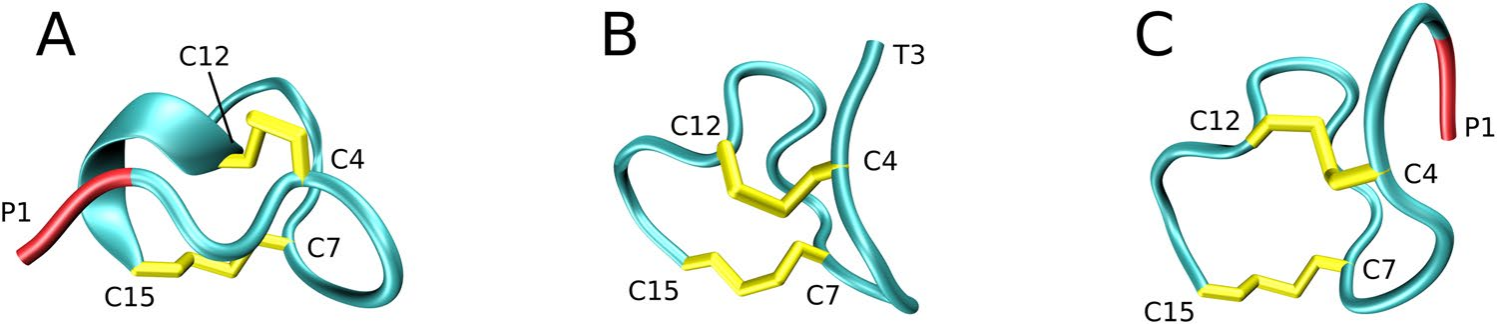
Structural models of isomer 2(**B**) of guanylin. (**A**) Low-energy MD model (marked by straight green arrow in Fig. 6). Shown is 1 conformer out of 20 that has the lowest GBSA energy. (**B**) NMR model 1GNB^70^. Shown is the conformer #10. (**C**) High-energy 1GNB-like MD model (marked by wavy green arrow in Fig. 6). Shown is 1 conformer out of 20 that has the lowest backbone *rmsd* to one of the 1GNB conformers (specifically, to the conformer #10). Disulfide bridges are painted gold; two N-terminal residues which are a part of the MD coordinates, but absent from the NMR construct, are painted red. The three structures have been superimposed prior to plotting to ensure that they are shown in the same perspective.

Considering the standard quality metrics, it appears that the low-energy MD model is actually better than the NMR structure. In principle, it would be desirable to validate the MD model against the experimental NOE data; however, these data are unavailable for the deposition 1GNB. Therefore we turn directly to the atomic coordinates. As it turns out, the MD and NMR coordinates are rather dissimilar. The MD model in Fig. 7A features a short 3_10_-helix comprising residues 12–14. The C-terminus of this helix is capped by the N-terminal tail of the peptide. Specifically, the C-terminal carboxylic group from residue C15 forms a stable salt bridge with the N-terminal amino group of residue P1 and, additionally, engages in hydrogen bonds with the side-chain hydroxyl of residue T3, as well as backbone amides of T3 and G2. There are also intermittent hydrogen bonds involving G2 and T3 as donors with C12 and T13 as acceptors. None of these features are found in the NMR structure 1GNB, where the N-terminal portion of the peptide is positioned differently, see Fig. 7B.

What is the reason for this disparity? As it happens, the NMR structure 1GNB of guanylin has been solved using a truncated version of guanylin sequence missing the first two N-terminal residues. The sequence was shortened intentionally to simplify the homonuclear spectra from the sample containing a mixture of 2(A) and 2(B) species^70^. It appears, however, that the absence of the two N-terminal residues has compromised the structure. Indeed, the NMR model of truncated guanylin 1GNB has very high C^α^ *rmsd* of 1.36 Å (see Table 2). For comparison, its counterpart structure 1GNA, which represents the isomer 2(A) and has been solved using the same NMR dataset, has *rmsd* of 0.44 Å. We conclude that the truncated 13-aminoacid guanylin in the form 2(B) is poorly structured and largely disordered (even though it is partially constrained by a pair of disulfide bonds). This is reflected in the low quality of its NMR structure.

**Table 2:** C^α^ coordinate variance for MD and NMR models of isomer 2(B) of guanylin, where each model is an ensemble of 20 conformers.

		Number of residues	Ensemble *rmsd*, Å	Lowest pairwise *rmsd*, Å		
				LeMD	NMR	HeMD
Low-energy MD model **LeMD**		15	0.36		2.41	4.18
NMR structure 1GNB **NMR**		13	1.36			1.39
High-energy 1GNB-like MD model **HeMD**		15	1.09			

The calculations of *rmsd* involve either thirteen or fifteen C^α^ atoms, depending on the number of residues in the analyzed models.

On the other hand, the physiologically relevant 15-aminoacid guanylin in the form 2(B) appears to be nicely structured according to the MD-based predictions. The obtained low-energy MD model is highly stable, with C^α^ *rmsd* of just 0.36 Å (calculated for the ensemble comprised of 20 conformers). It is worth noting that another low-energy model obtained in our simulations (cf. Fig. 6) essentially replicates this result. At the same time, the low-energy MD model is quite distinct from 1GNB coordinates, with pairwise *rmsd* exceeding 2.41 Å. It remains to be seen whether the predicted structure of full-length guanylin can be confirmed experimentally.

Finally, it is worth mentioning that among our MD data we also find one trajectory which resembles the NMR conformation 1GNB. Energetically, this is the least favored state of all realizations of isomer 2 (indicated by the wavy green arrow in Fig. 6). The 20-strong ensemble derived from this particular trajectory is in good agreement with 1GNB (minimal pairwise *rmsd* 1.39 Å), conformationally diverse (ensemble *rmsd* 1.09 Å) and clearly distinct from the well-folded 2(B) species (minimal pairwise *rmsd* 4.18 Å). We conclude that 1GNB-like conformation can also be encountered for the full-length guanylin, but rather as short-lived minor species and not as a stable fold corresponding to the global free-energy minimum.

### Kinetic vs. thermodynamic control

The reduced form of guanylin is disordered and does not adopt a stable fold, even though it produces transient elements of structure. The disordered nature of guanylin determines the character of cysteine-cysteine interactions – the frequency of thiol encounters depends on the separation in the primary sequence between the cysteines, steric hindrances in a form of side chains, local conformational preferences, etc. Only after disulfide bridges are formed, the peptide adopts a certain distinctive fold. However, the folded form of guanylin is likely quite different from the conformation that gives rise to the first disulfide bridge. Therefore it can be said that oxidative folding of guanylin occurs with the elements of kinetic control – disulfide bonding happens in the disordered state and does not necessarily correspond to the folded isomer with the lowest free energy.

Our MD simulations are in broad agreement with the above concept. During the equilibration period and until the disulfide bridges are formed the peptide remains disordered. Although it adopts various compact conformations, these conformations are never very stable. The cysteines are paired as dictated by the internal dynamics of the disordered peptide – with little correlation to the free energy of the resulting folded species. As a consequence, the simulations frequently produce high-energy isomers 1 and 3, whereas the low-energy isomer 2 turns out to be relatively rare (see Fig. 6).

It is interesting to observe that the relatively straightforward and even crude MD model can reasonably well reproduce the experimental isomer distribution that stems from the folding process under kinetic control. In our particular situation, kinetic control involves thiol encounters in the disordered peptide that are mostly governed by simple factors such as the sequence separation between cysteines and the bulkiness of the intervening amino acids. On the other hand, thermodynamic control reflects fine balance between multiple enthalpic and entropic contributions as found in the fully folded peptide species. One may speculate that it is generally easier for MD simulations to reproduce the folding behavior under kinetic control than thermodynamic control. It is clear, however, that such hypothesis needs further testing and investigation.

### Simulations of guanylin with adjusted thiol reactivity

Generally, there are two characteristic time scales in proteins undergoing oxidative folding: one associated with conventional folding (driven by hydrophobic effect, conformational propensities, etc.) and the other associated with disulfide bonding. While it may not be possible to reproduce the absolute rate of disulfide formation in an MD model (e.g. the reaction is too slow), it is important to maintain a proper balance between the rates of disulfide formation vs. the other mechanisms of folding. For example, in the guanylin simulations described above each thiol encounter is treated as productive. It is a simple matter to adjust the reaction rate downward such as to ensure that disulfide bonding is slow compared to the conformational dynamics of guanylin. This can be seen as a sufficient condition for an MD model of oxidative folding to be broadly valid.

To illustrate this point, we have implemented a simple computational strategy based on rejection sampling (a simple version of Monte-Carlo sampling). In essence, we postulate that there is a certain probability *p*_0_ that two proximal thiols would form a disulfide bond over the time interval of 1 ps. Technically, at every step where two sulfur atoms are found within 2.5 Å of each other we generate a random number *x* that is uniformly distributed between 0 and 1. If *x* happens to be less than the threshold value *p*_o_ = 0.001, then the procedure is launched to form a disulfide bridge; otherwise no action is taken and the simulation is continued. Using this modified protocol, we have recorded an additional series of 50 guanylin trajectories. Out of this number, 34 trajectories successfully formed both disulfide bridges within 1 μs oxidative folding period; each of these productive simulations was subsequently extended by 450-ns conventional MD run. All of the remaining trajectories have formed a single disulfide bond.

For the specific setting of *p*_o_ = 0.001 used in these simulations, the average time of formation of the first disulfide bond turns out to be 20 ns. Recall that in our original model corresponding to *p*_0_ = 1.0 the equivalent number has been 5 ns. Thus the process of forming disulfide bonds is slowed down several-fold when using the modified protocol (see Fig. 5B for complete kinetic footprint). Why is not it slowed down more significantly? As it happens, the formation of disulfide bond is typically preceded by guanylin adopting a certain conformation that is conducive to disulfide bonding. Such conformers are typically characterized by sulfur-sulfur distances fluctuating in the range ca. 2–4 Å and relatively long lifetime, up to at least ten nanoseconds. Because of the long lifetime, such conformational states often turn out to be productive, i.e. lead to disulfide-bonded species. In other words, although the probability of forming a disulfide bond at each individual step is low, the oxidation nevertheless happens relatively quickly because thiols tend to remain in the vicinity of each other for sufficiently long periods of time.

If necessary, the setting of *p*_0_ can be further adjusted. In doing so, the objective is to ensure that the reaction rate is slow compared to guanylin conformational dynamics, but still sufficiently fast to complete the simulations within a reasonable time frame. At this point it is appropriate to comment on the meaning of Fig. 5. Evidently, the absolute rates of disulfide formation as observed in our simulations are of no interest since they are many orders of magnitude higher than the experimentally observed rates. However, we believe that the relative characteristics, e.g. average time of formation of the first disulfide bond vs. the second disulfide bond, are potentially meaningful and relevant. In particular, if one conducts a series of simulations with progressively lowered thiol reactivity, such relative indicators should converge to realistic values that are representative of the experimental system.

Finally, we briefly describe the outcome of the series of simulations with reduced thiol reactivity. The obtained isomer distribution for the fully oxidized guanylin species is 53%: 12%: 35% for isomers 1, 2 and 3, respectively. If we consider all 50 trajectories, including those trajectories that committed to a certain isomer type by forming only one disulfide bond, we find the isomer distribution 42%: 22%: 36%. This is very similar to the results from our original MD model, see Table 1. This suggests that the model is reasonably well converged with regard to the isomer production. It is worth mentioning that the series of simulations with reduced thiol reactivity produced two low-energy models of isomer 2. One of them is of 2(B) type and closely resembles the conformation shown in Fig. 7A, while the other is of 2(A) type and looks similar to the high-quality NMR structure 1GNA.

### Oxidative folding of guanylin within proguanylin

**Figure 8:**
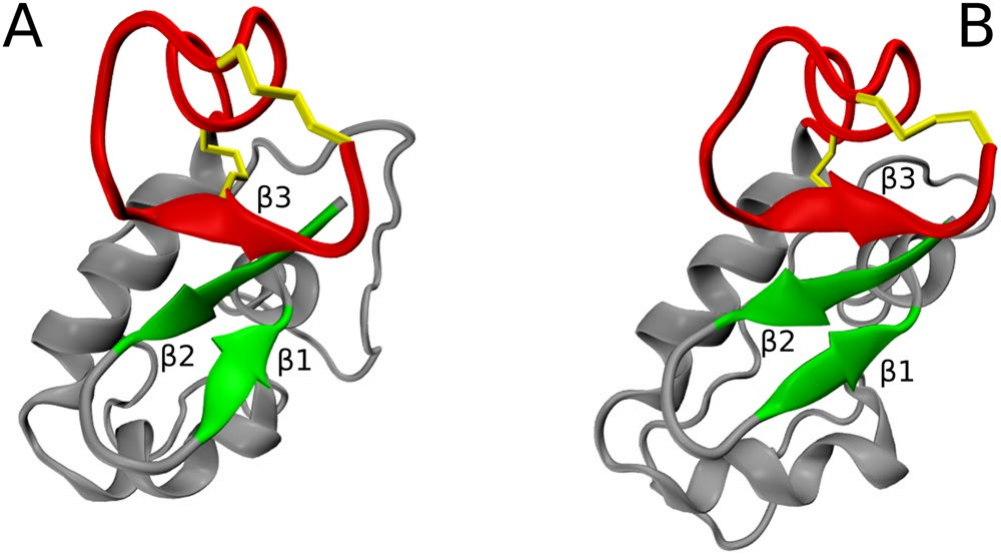
Structures of proguanylin from (**A**) NMR model 1O8R^74^ and (**B**) MD model of oxidative folding. The NMR model consists of 30 conformers; similarly, the MD model is built from 30 frames which have been extracted from the final portion of the trajectory (sampling step 1 ns). Shown in the plot are two conformers, one from the NMR model and one from the MD model, chosen on the basis of their favorable pairwise *rmsd* (see text). The guanylin segment, including β-sheet strand β3, is painted red; the two other strands that belong to the body of proguanylin, β1 and β2, are painted green. Note that the secondary-structure scaffold of proguanylin remains stable throughout the simulation, whereas long loops in the body of the protein experience significant motion.

*In vivo*, guanylin undergoes oxidative folding as a part of prohormone proguanylin. Proguanylin is a 94-aminoacid globular protein where guanylin sequence comprises the last fifteen C-terminal residues. NMR structure of proguanylin has been solved by Lauber *et al*. (PDB ID 1O8R, illustrated in Fig. 8A)^74^. The inspection of the structure reveals that guanylin “tail” is packed against the “body” of proguanylin, forming the extension of the small β-sheet. Under these conditions, the tail adopts the physiologically relevant conformation 2(A). After proguanylin is secreted in the intestine, it undergoes proteolytic cleavage, releasing the active form of guanylin into the circulation (the details of this mechanism are under active investigation)^75^.

The existing experimental evidence on the oxidative folding of guanylin^67^ and proguanylin^74, 76, 77^ provides a good insight into the role of the prohormone. As it appears, the body of proguanylin folds first; the folded body provides a template for subsequent oxidative folding of the guanylin tail. This model offers an interesting opportunity to simulate oxidative folding of guanylin in a native-like environment.

Toward this end, we have set up the MD simulations of proguanylin. The folded body of proguanylin was taken from the NMR coordinates 1O8R. The guanylin tail has been generated with a random conformation, as described earlier, and then fused to the body. The resulting proguanylin models were solvated using similar tactic as previously used for guanylin. Specifically, we have prepared 1,000 proguanylin models, solvated them, and determined the maximum size of the obtained water box. This maximum size, 75 Å, was subsequently used in all proguanylin simulations (to create sufficient room for the disordered guanylin tail). Each simulation consisted of 50 ns equilibration period, followed by 50 ns oxidative folding period during which disulfide bonds were formed. Out of sixty-three such trajectories, one produced the fully formed isomer 2 containing two disulfide bonds. This successful simulation was subsequently extended by recording 0.45 μs conventional MD trajectory.

The inspection of the productive proguanylin trajectory reveals that guanylin tail, which is initially in a random conformation, at around 10 ns forms an extension of the small β sheet in the protein structure (strand β3, see Fig. 8). Two pairs of cysteins thereby become aligned, favoring disulfide bonding according to the isomer 2(**A**) pattern. Consequently, the disulfides are formed at 54 and then 56 ns into the simulation. The resulting fold, which closely resembles the original structure 1O8R, undergoes a series of local adjustments, stabilizes at ca. 108 ns and after that remains essentially unchanged until the end of the simulation.

Shown in Fig. 8 is the comparison between the NMR model and the model extracted from the MD trajectory of proguanylin. When the two structures are overlaid by superimposing the secondary-structure portion of the proguanylin body, the guanylin tail segments are found to be within 2.3 Å (C^α^ atoms) of each other. When the tails are superimposed directly, the residual *rmsd* amounts to mere 1.1 Å (C^α^ atoms). These numbers suggest that the local structure of the guanylin “domain” is reproduced in the MD simulation with remarkable accuracy, but the positioning of the guanylin relative to the body of proguanylin is a bit less accurate. Careful inspection of the coordinates confirms that this is indeed the case. The structure of guanylin domain is in excellent agreement with 1O8R and remains stable beginning from 108 ns and until the end of the simulation. At the same time we observe a slight bending of the β-sheet, with strands β2 and particularly β3 “sliding back” during the course of the MD simulation. As a result, the guanylin domain becomes displaced by several angstroms relative to its position in 1O8R.

**Figure 9:**
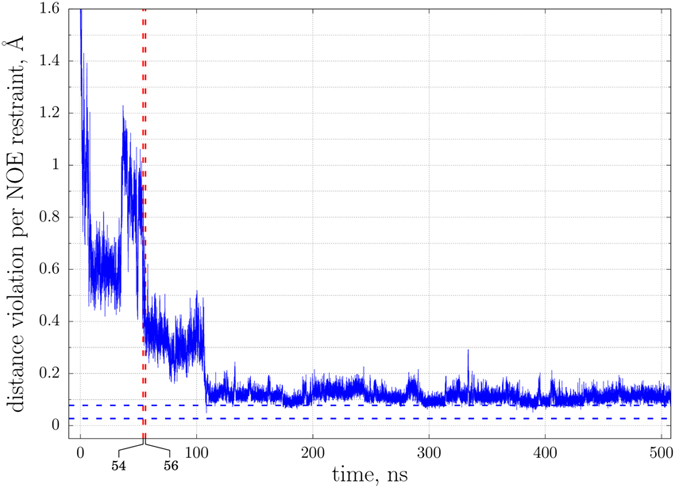
The magnitude of NOE violations observed in the MD simulation of oxidative folding of proguanylin (continuous line) and in the proguanylin structure 1O8R (dashed horizontal lines representing the minimum/maximum magnitude of violations in the NMR ensemble). Only those NOE restraints originating at the atoms in the guanylin tail have been included in the calculations. The moments when disulfide bridges are formed are indicated by red vertical lines.

In order to obtain more direct insight into the quality of the MD model, we have also tested it against the experimental NOE data that are available as a part of 1O8R PDB deposition. Because our MD simulation aims to model the folding of guanylin tail on the pre-folded proguanylin body, only tail-to-tail and tail-to-body NOE contacts have been included in the analyses. The results are presented in Fig. 9. The continuous profile in the plot indicates the time-dependent magnitude of NOE violations during the course of the MD trajectory. The initial MD coordinates containing randomly configured tail show large discrepancy with the experimental NOE data. As the simulation progresses and the tail becomes folded, the magnitude of NOE error decreases and eventually reaches the plateau at around 0.11 Å per restraint.

The conformational state reached in the MD simulation generally does not fit the NOE data quite as well as the NMR ensemble, where the average error amounts to 0.04 Å per restraint. Note, however, that there are multiple individual MD frames that are characterized by the same level of accuracy as NMR conformers (corresponding to the points in Fig. 9 where the blue profile enters the corridor between the two dashed horizontal lines). Furthermore, to properly judge the quality of the MD model vs. the NMR ensemble, one should bear in mind two considerations. First, the NMR structure has been optimized against the NOE restraints, whereas the MD trajectory has been recorded with no reference to the NOE data. Second, one cannot expect of the MD model to be a perfect replica of the NMR structure.

The latter point deserves a separate discussion. To investigate the convergence properties of the MD solution, we have recorded 0.5-μs-long control trajectory starting from the coordinates 1O8R. As it turns out, in this simulation the structure of proguanylin quickly evolves into conformational state where the NOE error amounts to ca. 0.13 Å per restraint (see Fig. S5). Further analysis has shown that this state is similar to the one illustrated in Fig. 8B. Therefore two independent MD simulations started from different initial coordinates converge to one and the same structure, which is similar but not identical to the NMR structure. This observation is consistent with the recent findings of Raval *et al*. who concluded that “global free-energy minimum of the force field… is different from the X-ray or NMR structure”^78^. In this connection, one should keep in mind that MD models are not necessarily wholly inferior to the experimental structures. While crystallographic coordinates are typically highly accurate, they can be influenced by crystal packing^79^. On the other hand NMR structures are generally less well determined and less accurate, in some extreme cases approaching the level of MD models^80^.

## Conclusion

In conventional MD simulations one makes an assumption that atomic composition of a protein molecule remains unchanged throughout the trajectory. Oftentimes this assumption proves to be restrictive. A number of algorithms have been developed to circumvent this fundamental limitation. For example, methods for variable pH simulations have been actively pursued^46–48^. The proposed algorithm to include disulfide formation in protein MD simulations falls in the same category. It relies on a highly simplified view of the disulfide chemistry, resulting in a protocol that is uncomplicated and robust. Essentially the entire time (with the exception of short intervals when cysteine thiol groups come into close contact) the simulation proceeds under control of the force field which is not different from the original, unmodified force field. Even when the system is briefly exposed to the altered force field, the alterations are minimal (e.g. only the LJ interaction between the two specific atoms is edited). The outcome is the disulfide-bonded protein structure, which is not different from a standard MD model.

The proposed algorithm allows for certain relatively straightforward extensions. For instance, in the case when individual cysteine pK_a_’s are significantly different, one can introduce a set of reaction rate constants specific for different cysteine pairs. This can be accomplished by means of the rejection sampling scheme, similar to the one described in this paper. Specifically, success rates for encounters involving different cysteine pairs can be assigned according to their specific pK_a_ values. Note, however, that such a scheme should be based on time-averaged rather than instantaneous pK_a_ values (recall that pK_a_’s are linked to formation of reaction intermediates and not the reaction rate itself).

For samples containing glutathione / oxidized glutathione, it is also possible to introduce an option for breaking disulfide bonds. It can be stipulated that at any point in the trajectory there is a certain (low) probability *p*_*r*_ that disulfide bond becomes reduced. It can be further assumed that *p*_*r*_ is proportional to the solvent exposure of the given disulfide site (the intention is to account for the site’s accessibility to glutathione). The overall scaling for *p*_*r*_ values can be adjusted empirically based on the available experimental data. For example, it can be required that disulfide reduction is slow compared to oxidation; in this manner only the “failed” trapped states will be reduced and thus rescued. On a technical level, the same procedure that is used in this paper to model formation of disulfides can be reversed and used to model dissolution of disulfides. Along the same lines it is, in principle, possible to model thiol-disulfide exchange reactions in the context of protein folding/unfolding.

Both of the extensions described above are reminiscent of the constant-pH techniques, where the concept of pK_a_ plays a central role and protonation/deprotonation occurs as a reversible process. It is also worth noting that, similar to constant-pH simulations, our algorithm can be combined with various enhanced sampling schemes, such as replica exchange MD or accelerated MD^81, 82^. Such approach is clearly useful in the context of protein folding, and particularly so in the context of oxidative folding, where oxidation tends to be a limiting (slow) step. On the other hand, certain other options that are available in the constant-pH simulations cannot be easily implemented in the context of disulfide bonding. For example, use of the Metropolis Monte Carlo scheme to decide the outcome of cysteine encounters would likely be problematic. While in the constant-pH methods protonation/deprotonation can be viewed as a local perturbation with the well-defined energy cost^83, 84^, it is harder to quantify the effect of disulfide formation on the energy of the partially ordered peptide chain.

Ultimately, it can be envisaged that QM/MM methods^85^ will evolve into a tool to explore protein folding including formation of disulfide bonds. Such future calculations will include explicit reaction intermediates, as discussed above. However, currently such approach would be too cumbersome and expensive to implement in a context of a simple folding simulation, such as presented in this work.

*In silico* protein folding is computationally very expensive. It has only become feasible due to advent of powerful computers and the evolution of enhanced sampling schemes. Our MD algorithm complements these developments, allowing to also fold those proteins that contain disulfide bridges. In the case of peptides, viable MD models can be obtained in a short time frame using the widely available GPU computers, as demonstrated by our computational study of guanylin. At the same time our method should facilitate MD studies of folding intermediates as well as misfolded states that are contingent on certain disulfide bonding patterns^86–88^.

## Methods

Initial guanylin conformations have been generated using the software distributed through the server unfolded.uchicago.edu complemented with the program Scwrl4^56^. 100,000 random conformers have been solvated using Amber command *solvateOct* with *closeness* parameter set to 5 Å. The largest box size, as extracted from the footer of the resulting coordinate files, was *d* = 56 Å, where *d* is the diameter of the sphere inscribed in the truncated octahedron (*d* = *a* = *l*√6 where *a* is the parameter of the unit crystal cell and *l* is the length of the edge in the truncated octahedron). This size, *d* = 56 Å, has been used for all subsequent guanylin simulations. For this purpose, we have written a special script using *solvateOct* command where the *closeness* parameter is iteratively adjusted until the desired *d* is obtained.

Three cysteine residues in guanylin were initially assigned to the new CYR type. This new residue type was created using *ParmEd* program from the *AmberTools* suite. CYR cysteines are deprotonated and neutral; all parameters of residue type CYR are inherited from the disulfide-bonded type CYX (however, we choose not to create a disulfide bond for type CYR). The fourth cysteine, which is located at the C-terminus and carries net charge of −1.0 due to its carboxylic group, was assigned to the new type CCYR; it is modeled after the standard type CCYX. Elsewhere in the paper we refer to all reactive cysteines as CYR type, without making special reference to CCYR.

Aside from cysteine residues, the protonation state of guanylin was chosen assuming physiological pH (it is also in agreement with the experimental conditions used by Schulz *et al*.^67^, pH 8.3). The hydrated system was neutralized by adding a single Na^+^ ion and subjected to energy minimization (500 steps using harmonic restraints with force constant 200 kcal/mol·Å^2^, followed by 100 steps with no restraints), then heated from 0 to 293 K and equilibrated for 1 ns at 293 K. Following the heating stage, the size of the water box in the ensemble of 50 guanylin trajectories was found to be 53.50 ± 0.05 Å.

All subsequent simulations were conducted under Amber ff14SB force field in TIP3P water using the NPT ensemble. The modifications to the force field are described below. Langevin thermostat with collision frequency 2 ps^−1^ was employed to maintain constant temperature 293 K. A cutoff of 10.5 Å was used for nonbonded interactions; long-range electrostatic interactions were treated with the particle mesh Ewald method. All bonds involving hydrogen atoms were constrained using SHAKE algorithm. The integration step was 2 fs.

The guanylin simulations described in this paper consist of three stages: equilibration (100 ns), oxidative folding (variable length), and extension (450 ns). The first and the third stage are conducted under the standard ff14SB force field. In what follows we describe in detail the algorithm used during the second stage.

At the beginning of the second stage all pairwise LJ interactions between sulfur atoms from CYR residues are disabled. This allows sulfur atoms to move within the capture radius of each other. The simulation is conducted in 1-ns steps. Prior to the beginning of each step, a restart file is recorded. Following 1-ns MD execution run, protein coordinates are saved (with frames sampled at 1-ps step from *i*=1 to 1000). A specialized script is then called to analyze the generated 1-ns trajectory segment. If during this interval all CYR sulfur atoms stay away from each other (with pairwise distance greater than 2.5 Å at all times) then the simulation is continued and the next 1-ns fragment is recorded. Conversely, if 2.5 Å line was crossed, then the procedure to form a disulfide bridge is launched:

(*i*) The number of the frame *n* is determined where one of the sulfur-sulfur distances first dropped below 2.5 Å.

(*ii*) The restart file from the previous step is used to rerun a small portion of the simulation. Specifically, a part of the 1-ns segment recorded in the previous step is duplicated beginning with the frame *i*=1 and ending with the frame *n*. In this manner we reproduce the state of the system at the precise moment when 2.5 Å line is crossed.

(*iii*) The obtained state is used as a starting point to record the next 2-ns segment of the trajectory with artificial restraints imitating a disulfide bond. For example, the distance between the two sulfur atoms is restrained using the following harmonic potential, $\alpha {k}_{d}{({r}_{SS}-{r}_{SS}^{0})}^{2}$, where ${r}_{SS}^{0}=2.038\,\AA$ is the length of the disulfide bond as parameterized in the Amber force field, *k*_*d*_ = 166 kcal/mol/Å^2^ is the corresponding force constant, and *α* is the scaling factor, which is linearly increased from 0.0 to 1.0 during the 2-ns simulation segment (more specifically, *α* = *k*/200 with *k* initially set to 1 and then incremented every 10 ps). For complete information on restraints see Table S1.

(*iv*) After the 2-ns segment is completed, artificial restraints are replaced with (fully equivalent) generic disulfide bond. The two bonded CYR residues are reclassified into the standard CYX type (automatically, all LJ interactions of their sulfur atoms are returned to normal). The simulation is then continued along the same lines until the second disulfide bridge is formed.

All these steps are controlled by a master script, written in python. The master script invokes CUDA version of *pmemd* to record MD data; it also calls upon other python scripts, e.g. to analyze sulfur-sulfur distances. We note that in principle the well-established interfaces such as PLUMED^89^ or COLVARS^90^ can be adapted to service the protocol described in this paper.

The simulations were conducted using in-house GPU workstations in the Laboratory of Biomolecular NMR at SPbU. The production rate obtained in conventional guanylin simulations was 88 ns/day/card using NVIDIA GeForce GTX780 cards and 113 ns/day/card using Tesla K40m cards. The production rate during the oxidation folding stage was only marginally worse. Specifically, analysis of the 1-ns fragments of the MD trajectory slows down the progress of the simulation by only 0.6%. The additional time expense to form disulfide bridges correspond to 2–3 ns simulation per disulfide, which is insignificant for long trajectories such as described in this paper.

The protocol to model oxidative folding of guanylin with reduced CYR reactivity was implemented as follows. Similar to the standard protocol we analyze the latest 1-ns fragment of the trajectory to determine whether one of the sulfur-sulfur distances has dipped below 2.5 Å. For each frame within this fragment where *r*_*SS*_ ≤ 2.5 Å we generate a random number *x* that is uniformly distributed between 0 and 1. If *x* is less than *p*_0_ = 0.001 then the standard procedure to form a disulfide bond is initiated; otherwise no action is taken and we continue parsing the frames. If the current fragment is fully processed without triggering disulfide bonding, then the simulation is continued to generate the next 1-ns fragment, etc.

In addition we have also tested an alternative version of the algorithm where instead of disabling the pairwise LJ interactions between the reactive sulfur atoms we have changed the corresponding equilibrium distance *r*_0_ from 4 Å to 2 Å (cf. Fig. 1). Out of fifty simulations using this alternative protocol, thirty nine trajectories led to the fully oxidized guanylin species with the following isomer distribution: 35% of isomer 1, 23% of isomer 2, and 41% of isomer 3. Other findings from these simulations are similar to those described above and are not further discussed in this paper.

Proguanylin simulations were conducted in a similar manner to guanylin simulations. The initial models were built as described previously^91^. Briefly, random guanylin tails were “glued” to the body of proguanylin (first model in the structure 1O8R) by superimposing the peptide plane P80-G81. Note that the body contains pre-existing disulfide bridge C48-C61. The energies of the obtained models were evaluated using *sander* (option igb = 8). The list of models was then filtered to exclude those models that suffer from steric clashes and have energies outside the (approximate) normal distribution. The size of the proguanylin simulation was on average 29,700 atoms; the production rate was 47 ns/day/card on Tesla K40m cards.

To analyze compliance with the NOE data, we employed the following procedure. For each experimental NOE restraint, we assumed that: (*i*) the deviation is zero if the interatomic distance *r* extracted from MD or NMR coordinates is within the NOE range, ${r}_{low}^{NOE}$ to ${r}_{high}^{NOE}$; (*ii*) the deviation is $(r-{r}_{high}^{NOE})$ if *r* is greater than the upper bound ${r}_{high}^{NOE}$ and (*iii*) the deviation is $({r}_{low}^{NOE}-r)$ if *r* is less than the lower bound ${r}_{low}^{NOE}$. In the case of protons with unresolved spectral signals (e.g. methyl protons, indicated by the wildcard symbol in the NOE list) the distances were defined as $r={({\Sigma }_{i=1}^{n}{r}_{i}^{-6})}^{-1/6}$, where the summation is over all equivalent protons^92^. The calculated deviations were (linearly) combined and normalized, resulting in distance violation per single NOE restraint.

### Data availability

The complete collection of scripts and the MD trajectory files are available from the authors upon request.

## Electronic supplementary material

Supplementary Information: Simple MD-based model for oxidative folding of peptides and proteins

## References

[1] Duan Y, Kollman PA (1998). Pathways to a protein folding intermediate observed in a 1-microsecond simulation in aqueous solution. Science.

[2] Snow CD, Nguyen N, Pande VS, Gruebele M (2002). Absolute comparison of simulated and experimental protein-folding dynamics. Nature.

[3] Sorin EJ, Pande VS (2005). Exploring the helix-coil transition via all-atom equilibrium ensemble simulations. Biophys. J..

[4] Zhou RH, Berne BJ, Germain R (2001). The free energy landscape for beta hairpin folding in explicit water. Proc. Natl. Acad. Sci. USA.

[5] Garcia AE, Onuchic JN (2003). Folding a protein in a computer: an atomic description of the folding/unfolding of protein A. Proc. Natl. Acad. Sci. USA.

[6] Piana S, Lindorff-Larsen K, Shaw DE (2013). Atomic-level description of ubiquitin folding. Proc. Natl. Acad. Sci. USA.

[7] Piana S, Lindorff-Larsen K, Shaw DE (2013). Atomistic description of the folding of a dimeric protein. J. Phys. Chem. B.

[8] Sborgi L (2015). Interaction networks in protein folding via atomic-resolution experiments and long-time-scale Molecular Dynamics simulations. J. Am. Chem. Soc..

[9] Miao YL, Feixas F, Eun CS, McCammon JA (2015). Accelerated Molecular Dynamics simulations of protein folding. J. Comput. Chem..

[10] MacCallum JL, Perez A, Dill KA (2015). Determining protein structures by combining semireliable data with atomistic physical models by Bayesian inference. Proc. Natl. Acad. Sci. USA.

[11] Kobayashi Y, Sasabe H, Akutsu T, Saito N (1992). Mechanism of protein folding. 4. Forming and breaking disulfide bonds in bovine pancreatic trypsin inhibitor. Biophys. Chem..

[12] Camacho CJ, Thirumalai D (1995). Modeling the role of disulfide bonds in protein folding: entropic barriers and pathways. Proteins: Struct. Funct. Genet..

[13] Lu D, Liu Z (2008). Dynamic redox environment-intensified disulfide bond shuffling for protein refolding *in vitro*: molecular simulation and experimental validation. J. Phys. Chem. B.

[14] Martí-Renom NA, Stote RH, Querol E, Avilés FX, Karplus M (1998). Refolding of potato carboxypeptidase inhibitor by molecular dynamics simulations with disulfide bond constraints. J. Mol. Biol..

[15] Czaplewski C, Oldziej S, Liwo A, Scheraga HA (2004). Prediction of the structures of proteins with the UNRES force field, including dynamic formation and breaking of disulfide bonds. Protein Eng. Des. Sel..

[16] Kondov I, Verma A, Wenzel W (2009). Folding path and funnel scenarios for two small disulfide-bridged proteins. Biochemistry.

[17] Qin M, Zhang J, Wang W (2006). Effects of disulfide bonds on folding behavior and mechanism of the beta-sheet protein tendamistat. Biophys. J..

[18] Chinchio M, Czaplewski C, Liwo A, Oldziej S, Scheraga HA (2007). Dynamic formation and breaking of disulfide bonds in molecular dynamics simulations with the UNRES force field. J. Chem. Theory Comput..

[19] Shaw DE (2010). Atomic-level characterization of the structural dynamics of proteins. Science.

[20] Welker E, Narayan M, Wedemeyer WJ, Scheraga HA (2001). Structural determinants of oxidative folding in proteins. Proc. Natl. Acad. Sci. USA.

[21] Wedemeyer WJ, Welker E, Narayan M, Scheraga HA (2000). Disulfide bonds and protein folding. Biochemistry.

[22] Tu BP, Weissman JS (2004). Oxidative protein folding in eukaryotes: mechanisms and consequences. J. Cell Biol..

[23] Kosuri P (2012). Protein folding drives disulfide formation. Cell.

[24] Boudko SP, Engel J (2004). Structure formation in the C terminus of type III collagen guides disulfide cross-linking. J. Mol. Biol..

[25] Welker E, Wedemeyer WJ, Narayan M, Scheraga HA (2001). Coupling of conformational folding and disulfide-bond reactions in oxidative folding of proteins. Biochemistry.

[26] Dangoria NS (2002). HLA-B27 misfolding is associated with aberrant intermolecular disulfide bond formation (dimerization) in the endoplasmic reticulum. J. Biol. Chem..

[27] Niwa J (2007). Disulfide bond mediates aggregation, toxicity, and ubiquitylation of familial amyotrophic lateral sclerosis-linked mutant SOD1. J. Biol. Chem..

[28] Baneyx F, Mujacic M (2004). Recombinant protein folding and misfolding in Escherichia coli. Nat. Biotechnol..

[29] Moroder L, Besse D, Musiol HJ, Rudolph-Bohner S, Siedler F (1996). Oxidative folding of cystine-rich peptides vs regioselective cysteine pairing strategies. Biopolymers.

[30] Reinwarth M (2013). Oxidative folding of peptides with cystine-knot architectures: kinetic studies and optimization of folding conditions. Chembiochem.

[31] Belgi A, Hossain MA, Tregear GW, Wade JD (2011). The chemical synthesis of insulin: from the past to the present. Immunol. Endocr. Metab. Agents Med. Chem..

[32] Weiss MA (2013). Diabetes mellitus due to the toxic misfolding of proinsulin variants. FEBS Lett..

[33] Winterbourn CC, Hampton MB (2008). Thiol chemistry and specificity in redox signaling. Free Radical Bio. Med..

[34] Gupta V, Carroll KS (2014). Sulfenic acid chemistry, detection and cellular lifetime. *BBA Gen*. Subjects.

[35] Winterbourn CC, Metodiewa D (1999). Reactivity of biologically important thiol compounds with superoxide and hydrogen peroxide. Free Radical Bio. Med..

[36] Roos G, Messens J (2011). Protein sulfenic acid formation: From cellular damage to redox regulation. Free Radical Bio. Med..

[37] Rehder DS, Borges CR (2010). Cysteine sulfenic acid as an intermediate in disulfide bond formation and nonenzymatic protein folding. Biochemistry.

[38] Kassim R, Ramseyer C, Enescu M (2011). Oxidation of zinc-thiolate complexes of biological interest by hydrogen peroxide: a theoretical study. Inorg. Chem..

[39] Fernandes PA, Ramos MJ (2004). Theoretical insights into the mechanism for thiol/disulfide exchange. Chem. Eur. J..

[40] Poole LB, Karplus PA, Claiborne A (2004). Protein sulfenic acids in redox signaling. Annu. Rev. Pharmacol. Toxicol..

[41] Ferrer-Sueta G (2011). Factors affecting protein thiol reactivity and specificity in peroxide reduction. Chem. Res. Toxicol..

[42] Roos G, Foloppe N, Messens J (2013). Understanding the pKa of redox cysteines: the key role of hydrogen bonding. Antioxid. Redox Signal..

[43] Olah J, van Bergen L, De Proft F, Roos G (2015). How does the protein environment optimize the thermodynamics of thiol sulfenylation? Insights from model systems to QM/MM calculations on human 2-Cys peroxiredoxin. J. Biomol. Struct. Dyn..

[44] Kolberg M (2002). Protein thiyl radicals directly observed by EPR spectroscopy. Arch. Biochem. Biophys..

[45] Schoneich C (2008). Mechanisms of protein damage induced by cysteine thiyl radical formation. Chem. Res. Toxicol..

[46] Mongan J, Case DA, McCammon JA (2004). Constant pH molecular dynamics in generalized born implicit solvent. J. Comput. Chem..

[47] Lee MS, Salsbury FR, Brooks CL (2004). Constant-pH molecular dynamics using continuous titration coordinates. Proteins: Struct. Funct. Bioinf..

[48] Swails JM, York DM, Roitberg AE (2014). Constant pH replica exchange Molecular Dynamics in explicit solvent using discrete protonation states: implementation, testing, and validation. J. Chem. Theory Comput..

[49] Currie MG (1992). Guanylin: an endogenous activator of intestinal guanylate-cyclase. Proc. Natl. Acad. Sci. USA.

[50] Badock V, Raida M, Adermann K, Forssmann WG, Schrader M (1998). Distinction between the three disulfide isomers of guanylin 99-115 by low-energy collision-induced dissociation. Rapid Commun. Mass Spectrom..

[51] Shailubhai K (2000). Uroguanylin treatment suppresses polyp formation in the Apc^Min/+^ mouse and induces apoptosis in human colon adenocarcinoma cells via cyclic GMP. Cancer Res..

[52] Steinbrecher KA, Wowk SA, Rudolph JA, Witte DP, Cohen MB (2002). Targeted inactivation of the mouse guanylin gene results in altered dynamics of colonic epithelial proliferation. Am. J. Pathol..

[53] Carpick BW, Gariepy J (1993). The Escherichia Coli heat-stable enterotoxin is a long-lived superagonist of guanylin. Infect. Immun..

[54] Steinbrecher KA, Cohen MB (1999). Guanylin, uroguanylin and guanylate cyclase C: Regulation in a mouse model in osmotic diarrhea. FASEB J..

[55] Jha AK, Colubri A, Freed KF, Sosnick TR (2005). Statistical coil model of the unfolded state: Resolving the reconciliation problem. Proc. Natl. Acad. Sci. USA.

[56] Krivov GG, Shapovalov MV, Dunbrack RL (2009). Improved prediction of protein side-chain conformations with SCWRL4. Proteins: Struct. Funct. Bioinf..

[57] Nguyen H, Roe DR, Simmerling C (2013). Improved Generalized Born solvent model parameters for protein simulations. J. Chem. Theory Comput..

[58] Case, D. A. *et al*. AMBER 14. (University of California, 2014).

[59] Bryngelson JD, Wolynes PG (1987). Spin glasses and the statistical mechanics of protein folding. Proc. Natl. Acad. Sci. USA.

[60] Bryngelson JD, Onuchic JN, Socci ND, Wolynes PG (1995). Funnels, pathways, and the energy landscape of protein folding: a synthesis. Proteins: Struct. Funct. Genet..

[61] Best RB, Zheng WW, Mittal J (2014). Balanced protein-water interactions improve properties of disordered proteins and non-specific protein association. J. Chem. Theory Comput..

[62] Piana S, Donchev AG, Robustelli P, Shaw DE (2015). Water dispersion interactions strongly influence simulated structural properties of disordered protein states. J. Phys. Chem. B.

[63] Olsson MHM, Sondergaard CR, Rostkowski M, Jensen JH (2011). PROPKA3: consistent treatment of internal and surface residues in empirical pK_a_ predictions. J. Chem. Theory Comput..

[64] Kortemme T, Creighton TE (1995). Ionisation of cysteine residues at the termini of model α-helical peptides. Relevance to unusual thiol pKa values in proteins of the thioredoxin family. J. Mol. Biol..

[65] Tang SS, Chang GG (1996). Kinetic characterization of the endogenous glutathione transferase activity of octopus lens S-crystallin. J. Biochem..

[66] Bourles E, Isaac M, Lebrun C, Latour J-M, Seneque O (2011). Oxidation of Zn(Cys)_4_ Zinc Finger peptides by O_2_ and H_2_O_2_: products, mechanism and kinetics. Chem. Eur. J..

[67] Schulz A (1999). Role of the prosequence of guanylin. Protein Sci..

[68] Gohlke H, Case DA (2004). Converging free energy estimates: MM-PB(GB)SA studies on the protein-protein complex Ras-Raf. J. Comput. Chem..

[69] Genheden S, Ryde U (2015). The MM/PBSA and MM/GBSA methods to estimate ligand-binding affinities. Expert Opin. Drug Discov..

[70] Skelton NJ, Garcia KC, Goeddel DV, Quan C, Burnier JP (1994). Determination of the solution structure of the peptide hormone guanylin: observation of a novel form of topological stereoisomerism. Biochemistry.

[71] Schulz A (1998). Carboxy-terminal extension stabilizes the topological stereoisomers of guanylin. J. Pept. Res..

[72] Lovell SC (2003). Structure validation by Cα geometry: f, ψ and Cβ deviation. Proteins: Struct. Funct. Genet..

[73] Vriend G (1990). WHAT IF: a molecular modeling and drug design program. J. Mol. Graphics.

[74] Lauber T, Neudecker P, Rosch P, Marx UC (2003). Solution structure of human proguanylin. The role of a hormone prosequence. J. Biol. Chem..

[75] Valentino MA (2011). A uroguanylin-GUCY2C endocrine axis regulates feeding in mice. J. Clin. Invest..

[76] Lauber T, Schulz A, Rosch P, Marx UC (2004). Role of disulfide bonds for the structure and folding of proguanylin. Biochemistry.

[77] Lauber T, Marx UC (2005). Prosequence-mediated disulfide coupled folding of the peptide hormones guanylin and uroguanylin. Protein Pept. Lett..

[78] Raval A, Piana S, Eastwood MP, Dror RO, Shaw DE (2012). Refinement of protein structure homology models via long, all-atom molecular dynamics simulations. Proteins: Struct. Funct. Bioinf..

[79] Kossiakoff AA, Randal M, Guenot J, Eigenbrot C (1992). Variability of conformations at crystal contacts in BPTI represent true low-energy structures: correspondence among lattice packing and molecular dynamics structures. Proteins: Struct. Funct. Genet..

[80] Andrec M (2007). A large data set comparison of protein structures determined by crystallography and NMR: Statistical test for structural differences and the effect of crystal packing. Proteins: Struct. Funct. Bioinf..

[81] Swails JM, Roitberg AE (2012). Enhancing conformation and protonation state sampling of Hen Egg White Lysozyme using pH Replica Exchange Molecular Dynamics. J. Chem. Theory Comput..

[82] Williams SL, de Oliveira CAF, McCammon JA (2010). Coupling constant pH molecular dynamics with accelerated molecular dynamics. J. Chem. Theory Comput..

[83] Baptista AM, Teixeira VH, Soares CM (2002). Constant-pH molecular dynamics using stochastic titration. J. Chem. Phys..

[84] Chen YJ, Roux B (2015). Constant-pH hybrid nonequilibrium Molecular Dynamics Monte Carlo simulation method. J. Chem. Theory Comput..

[85] Rickard GA, Berges J, Houee-Levin C, Rauk A (2008). Ab initio and QM/MM study of electron addition on the disulfide bond in thioredoxin. J. Phys. Chem. B.

[86] Silvers R (2012). Modulation of structure and dynamics by disulfide bond formation in unfolded states. J. Am. Chem. Soc..

[87] Pucheta-Martinez, E. *et al*. Changes in the folding landscape of the WW domain provide a molecular mechanism for an inherited genetic syndrome. *Sci. Rep*. **6** (2016). 10.1038/srep30293 PMC4960638 27456546

[88] Zeiler RNW, Bolhuis PG (2015). Exposure of thiol groups in the heat-induced denaturation of beta-lactoglobulin. Mol. Simul..

[89] Tribello GA, Bonomi M, Branduardi D, Camilloni C, Bussi G (2014). PLUMED 2: new feathers for an old bird. Comput. Phys. Commun..

[90] Fiorin G, Klein ML, Henin J (2013). Using collective variables to drive molecular dynamics simulations. Mol. Phys..

[91] Yuwen T, Post CB, Skrynnikov NR (2011). Domain cooperativity in multidomain proteins: what can we learn from molecular alignment in anisotropic media?. J. Biomol. NMR.

[92] Nilges M, O’Donoghue SI (1998). Ambiguous NOEs and automated NOE assignment. Prog. NMR Spectrosc..

