# Supplementary material for: Simple MD-based model for oxidative folding of peptides and proteins
Source: Sci Rep. 2017 Aug 24;7:9293. doi: 10.1038/s41598-017-09229-7 (PMC5570944; doi:10.1038/s41598-017-09229-7)
Supplement: Supplementary file 1 — Supplementary Information: Simple MD-based model for oxidative folding of peptides and proteins [file 41598_2017_9229_MOESM1_ESM.pdf]

Simple MD-based model for oxidative folding of peptides and proteins

Sergei A. Izmailov,[1] Ivan S. Podkorytov,[1] and Nikolai R. Skrynnikov[1,2*]

[1] Laboratory of Biomolecular NMR, St. Petersburg State University, St. Petersburg 199034, Russia
[2] Department of Chemistry, Purdue University, West Lafayette IN 47907, USA

* Corresponding author. E-mail: nikolai@purdue.edu

	Atoms	Potential	Parameters
1	^{1}SG-^{2}SG	$k_d(d-d_0)^2$	k_d = 166 kcal/mol/Å^2, d_0=2.038 Å
2	^{1}CB-^{1}SG-^{2}SG	$k_\phi(\phi-\phi_0)^2$	k_ϕ = 68 kcal/mol/rad^2, ϕ_0=103.7°
3	^{1}SG-^{2}SG-^{2}CB		
4	^{1}CB-^{1}SG-^{2}SG-^{2}CB	$k_\theta(1+\cos(n\theta-\theta_0))$	k_θ =0.4200 kcal/mol, n=1, θ_0=0°
5	^{1}CB-^{1}SG-^{2}SG-^{2}CB		k_θ =4.4800 kcal/mol, n=2, θ_0=0°
6	^{1}CB-^{1}SG-^{2}SG-^{2}CB		k_θ =0.6820 kcal/mol, n=3, θ_0=0°
7	^{1}CB-^{1}SG-^{2}SG-^{2}CB		k_θ =0.3790 kcal/mol, n=4, θ_0=0°
8	^{1}SG-^{2}SG-^{2}CB-^{2}CA		k_θ =0.0560 kcal/mol, n=1, θ_0 =0°
9	^{1}CA-^{1}CB-^{1}SG-^{2}SG		
10	^{1}SG-^{2}SG-^{2}CB-^{2}CA		k_θ =0.6660 kcal/mol, n=2, θ_0=0°
11	^{1}CA-^{1}CB-^{1}SG-^{2}SG		
12	^{1}SG-^{2}SG-^{2}CB-^{2}CA		k_θ =0.3020 kcal/mol, n=3, θ_0=0°
13	^{1}CA-^{1}CB-^{1}SG-^{2}SG		
14	^{1}SG-^{2}SG-^{2}CB-^{2}CA		k_θ =0.1350 kcal/mol, n=4, θ_0=180°
15	^{1}CA-^{1}CB-^{1}SG-^{2}SG		
16	^{1}SG-^{2}SG-^{2}CB-^{2}HB2		k_θ =0.3333 kcal/mol, n=3, θ_0=0°
17	^{1}SG-^{2}SG-^{2}CB-^{2}HB3		
18	^{1}HB3-^{1}CB-^{1}SG-^{2}SG		
19	^{1}HB2-^{1}CB-^{1}SG-^{2}SG		

Table S1. Amber 14SB force field parameters associated with disulfide bond. The heavy atoms in the disulfide bond are labeled ^{1}CA-^{1}CB-^{1}SG-^{2}SG-^{2}CB-^{2}CA. A set of artificial restraints imitating (nascent) disulfide bond includes all of these terms with their respective force constants multiplied by the scaling factor α. The factor α is linearly increased from 0.0 to 1.0 during the 2-ns interval whereby the disulfide bond is established (more precisely, $\alpha = k/200$ with k initially set to 1 and then incremented every 10 ps).

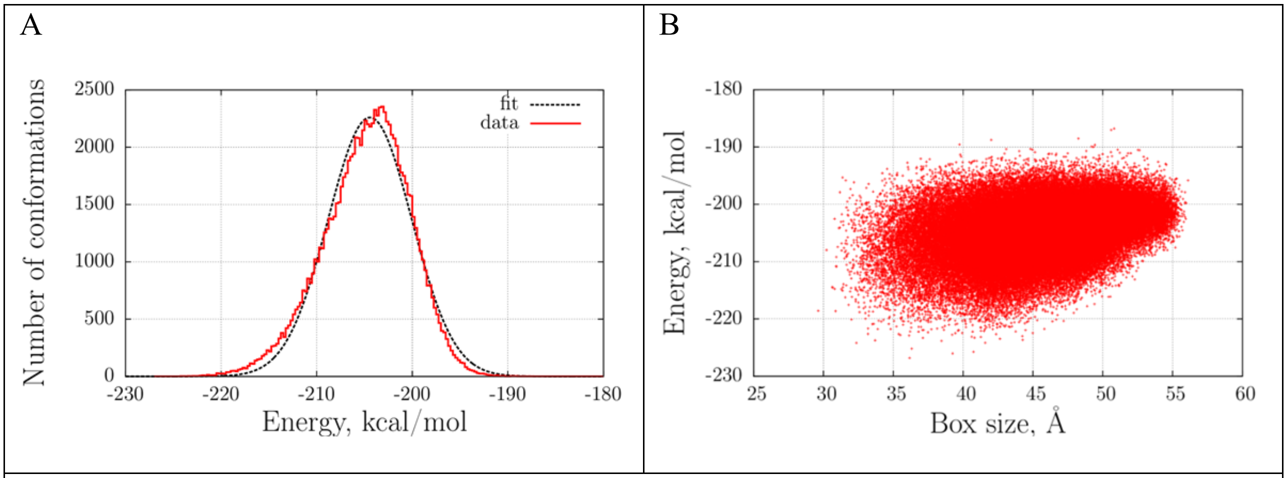

Figure S1. (**A**) Energy distribution of 100,000 random guanylin structures calculated using sander program in AmberTools with implicit solvent (ff14SB, igb=8). The histogram with the step 0.25 kcal/mol is shown with solid red line; the Gaussian approximation with the best-fit parameters $\mu = -204.4$ kcal/mol, $\sigma = 4.4$ kcal/mol is shown with black dashed line. (**B**) The energies of 100,000 random guanylin structures vs. the size of the water box as generated by solvateOct command in LEaP. The box size is the diameter d of the sphere inscribed into the truncated octahedron (which is related to the tetrahedron edge length l as $d = \sqrt{6}l$). Note that extended structures that require larger boxes seem to be somewhat less favorable energetically, which explains the "compaction" effect observed in Fig. 3.

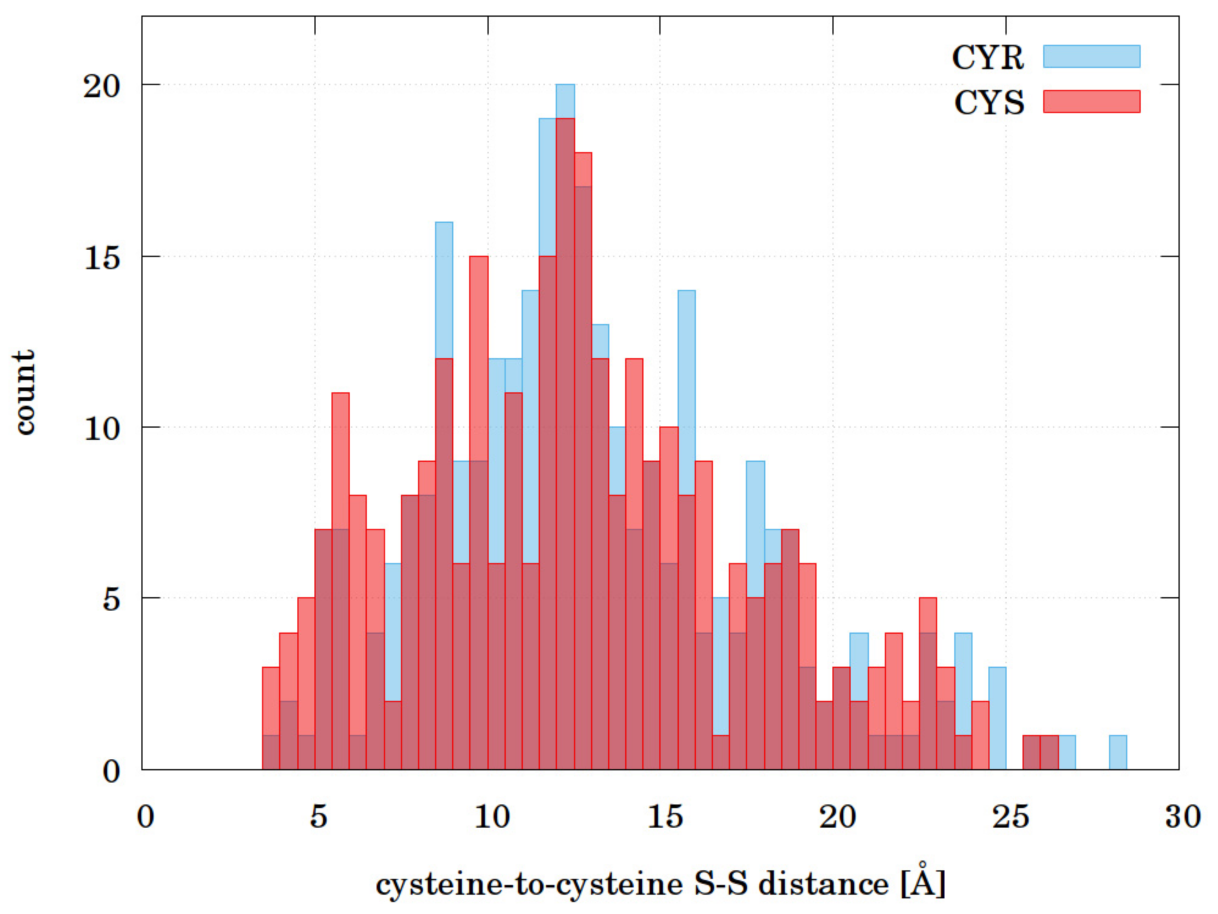

Figure S2. Histograms of cysteine-to-cysteine sulfur-sulfur distances in two conformational ensembles of guanylin. Each ensemble represents the final state in the series of fifty 100-ns trajectories that started from random peptide configurations; each of the fifty guanylin conformers is used to extract six pairwise sulfur-sulfur distances. The first series of trajectories is part of the simulations described in the main text where guanylin contains four deprotonated neutral CYR residues (cyan histogram). The second series of trajectories is control simulations where guanylin contains four standard CYS residues (pink histogram). The distance distributions are overall similar, indicating that the use of CYR residues does not lead to any undesirable packing effects. The average radii of gyration for the two respective ensembles are 7.55 and 7.47 Å (cf. Fig. 3).

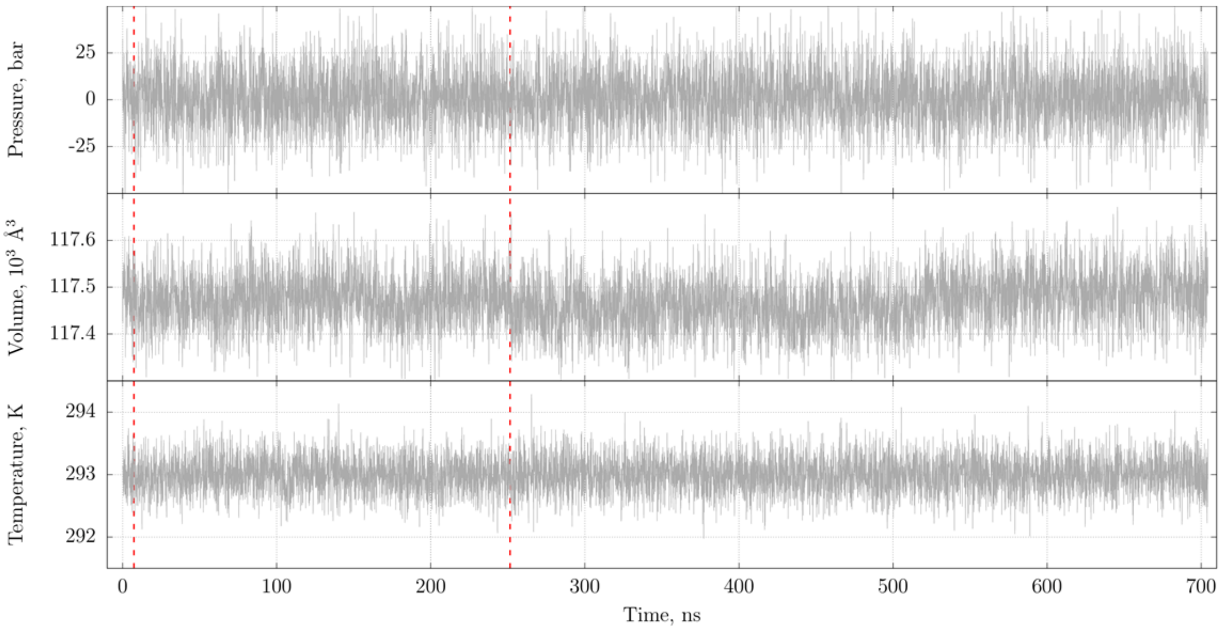

Figure S3. Time trace of pressure, volume, and temperature for the MD trajectory leading to the formation of low-energy isomer 2(B) of guanylin (see text). The moments when disulfide bridges are formed (i.e. the moments when sulfur atoms approach to within 2.5 Å thus triggering the process of disulfide formation) are indicated by vertical dashed lines. The graph covers (*i*) 253-ns oxidative folding stage followed by (*ii*) 450-ns evolution.

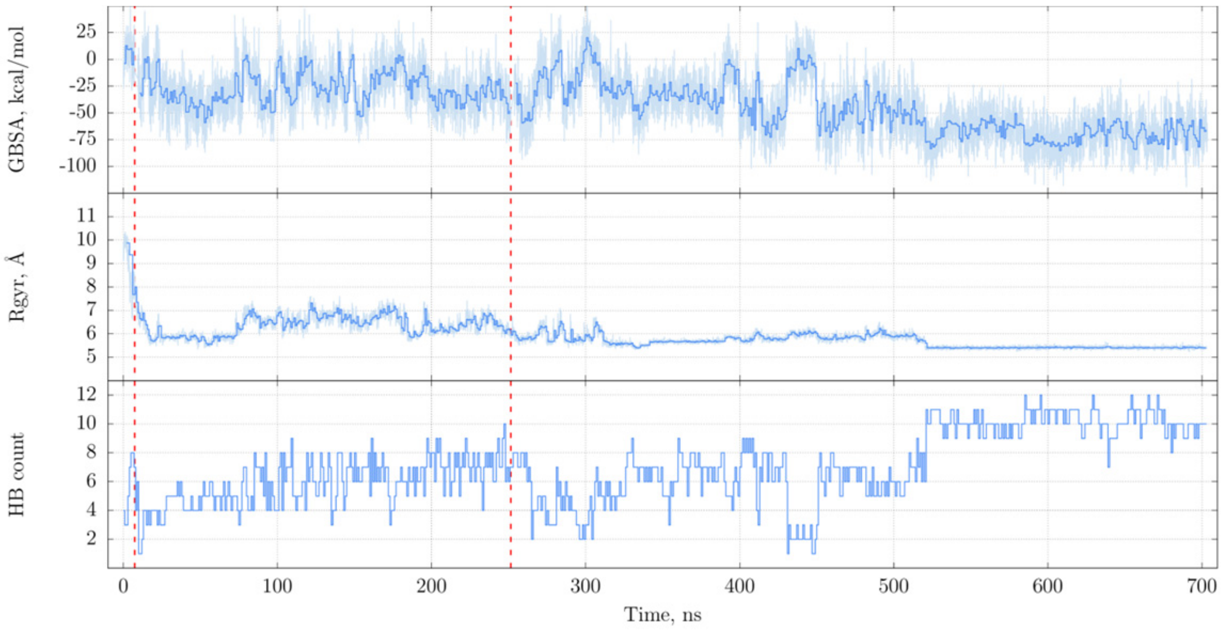

Figure S4. Time trace of GBSA free energy (igb=8), radius of gyration, and the number of intramolecular hydrogen bonds for the MD trajectory leading to the formation of low-energy isomer 2(B) of guanylin (see text). The GBSA energy is shown throughout the course of the trajectory except 2-ns intervals where artificial restraints are used to emulate nascent disulfide bonds. To determine hydrogen bond content, we divided the trajectory into 1 ns segments. Within each segment we have analyzed 100 frames (sampling step 10 ps). If given hydrogen bond is registered in at least half of these frames then it is included in the current count. Hydrogen bonds were identified using the program *hbplus* [I.K. McDonald and J.M. Thornton (1994) *J. Mol. Biol.*, **238**, 777].

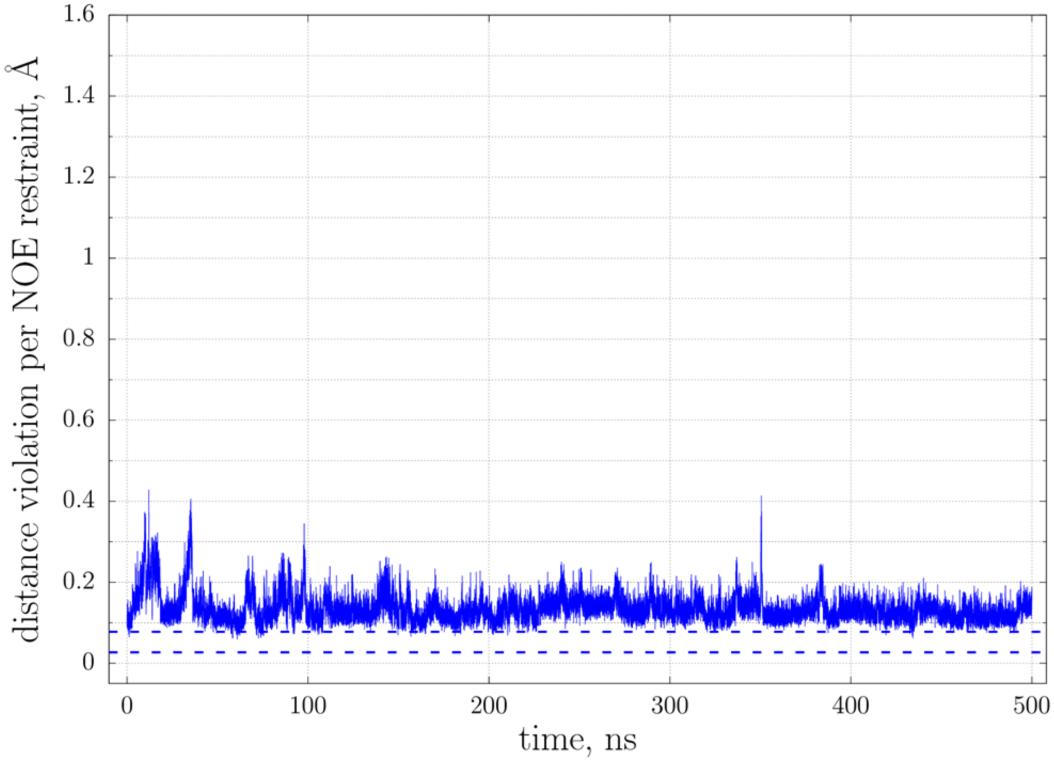

Figure S5. The magnitude of NOE violations observed in the MD simulation of proguanylin and in the NMR structure 1O8R. The starting coordinates of the MD simulation are those of the first conformer in 1O8R. Plotting conventions are the same as in Fig. 9.
